# Saudi National Clinical Practice Guidelines for Management of Adult Systemic Lupus Erythematosus

**DOI:** 10.2174/0115733971275638240429063041

**Published:** 2024-04-30

**Authors:** Ahmed H. Al-Jedai, Hajer Y. Almudaiheem, Ibrahim A. Al-Homood, Ibrahim Almaghlouth, Sami M. Bahlas, Abdulaziz Mohammed Alolaiwi, Mohammad Fatani, Maysa Tariq Eshmawi, Bedor A. AlOmari, Khalidah Ahmed Alenzi, Rayan G. Albarakati, Nayef Al Ghanim

**Affiliations:** 1 Deputyship of Therapeutic Affairs, Ministry of Health, Riyadh, Saudi Arabia;; 2 Colleges of Medicine and Pharmacy, Al Faisal University, Riyadh, Saudi Arabia;; 3 Medical Specialties Department, King Fahad Medical City, Riyadh, Saudi Arabia;; 4 Medicine Department, College of Medicine, Al Faisal University, Riyadh, Saudi Arabia;; 5 Department of Medicine, College of Medicine, King Saud University, Riyadh 11461, Saudi Arabia;; 6 College of Medicine Research Center, King Saud University, Riyadh 11461, Saudi Arabia;; 7 Department of Internal Medicine, Faculty of Medicine, King Abdulaziz University, Jeddah 21589, Saudi Arabia;; 8 Department of Rheumatology, King Saud Medical City, Riyadh, Saudi Arabia;; 9 Hera General Hospital, Ministry of Health, Makkah, Saudi Arabia;; 10 King Abdullah Medical Complex, Jeddah, Saudi Arabia;; 11 College of Medicine, Imam Mohammad Ibn Saud Islamic University, Riyadh, Saudi Arabia;; 12 Department of Pharmaceutical Services, Prince Sultan Military Medical City, Riyadh, Saudi Arabia;; 13 Tabuk Health Cluster, Tabuk, Saudi Arabia;; 14 Department of Obstetrics and Gynecology, Majmaah University, Al-Majmaah 11952, Saudi Arabia;; 15 Department of Rheumatology, King Saud Medical City, Riyadh, Saudi Arabia

**Keywords:** Systemic lupus erythematosus, disease activity, guidelines, Saudi Arabia, Steroid-sparing agent, glucocorticoid

## Abstract

**Objective:**

To provide evidence-based clinical practice recommendations for managing Systemic Lupus Erythematosus (SLE) in Saudi Arabia.

**Methods:**

This EULAR-adapted national guideline in which a multidisciplinary task force utilized the modified Delphi method to develop 31 clinical key questions. A systematic literature review was conducted to update the evidence since the EULAR publication. After reaching a consensus agreement, two rounds of voting and group discussion were conducted to generate consolidated recommendations/statements.

**Results:**

A significant number of patients in Saudi Arabia experience delays in accessing rheumatologists, highlighting the significance of timely referral to SLE specialists or rheumatologists to ensure accurate diagnosis and prompt treatment. The primary goal of Glucocorticoid (GC) therapy in SLE patients is to establish disease control with a minimum dose and duration. Steroid-sparing agent utilization facilitates steroid-sparing goals. Hydroxychloroquine is recommended for all SLE patients, though physicians must carefully monitor toxicity and prioritize regular medication adherence assessment. SLE management during pregnancy starts from preconception time by assessing disease activity, major organ involvement, hypercoagulability status, and concomitant diseases that may negatively impact maternal and fetal outcomes. Multidisciplinary care with close monitoring may optimize both maternal and fetal outcomes. For patients with antiphospholipid antibodies, low-dose aspirin prophylaxis is recommended. Also, Long-term anticoagulant medications are fundamental to prevent secondary antiphospholipid syndrome due to high thrombosis recurrence.

**Conclusion:**

This Saudi National Clinical Practice guidelines for SLE management provide evidence-based recommendations and guidance for healthcare providers in Saudi Arabia who are managing patients with SLE. These guidelines will help to standardize healthcare service, improve provider education, and perhaps lead to better treatment outcomes for SLE patients.

## 1 INTRODUCTION

Systemic Lupus Erythematosus (SLE) is an autoimmune disease affecting various body organs and systems [1]. It is characterized by autoantibodies that are produced against self-antigens, causing deposition of the immune complexes followed by inflammation and organ tissue damage [1]. SLE has a variable course and wide presentations, with periods of flares and remission [2, 3]. Due to its chronic nature and potentially life-threatening complications, it poses a significant burden on patients, families, and healthcare systems worldwide.

The management of SLE requires a systematic approach and a tailored treatment plan based on the individual patient's disease activity, organ involvement, complications, and associated comorbidities. The current understanding of SLE pathogenesis and the availability of novel therapeutic options have led to significant advancements in the management of SLE.

In Saudi Arabia, the prevalence of SLE is estimated to be around 19 cases per 100,000 individuals, with a higher incidence in women and young adults [4]. The burden of SLE on patients and healthcare systems is significant, with a high risk of morbidity and mortality and decreased Quality of Life (QoL) [5]. Furthermore, the rapid development of new therapeutics and the rising cost of such therapies underscore the necessity for standardized and evidence-based recommendations in managing SLE.

This paper aims to provide the Saudi national clinical practice recommendations for SLE management, highlighting the key recommendations and their rationale. It also discusses the implications of these recommendations for clinical practice, research, and healthcare policymaking in Saudi Arabia and the Middle East. These evidence-based clinical practice recommendations are expected to improve the quality of care and treatment outcomes for SLE patients in Saudi Arabia and serve as a crucial resource for clinicians and researchers in the Middle East and worldwide.

## 2 METHODS

**Fig. (1):**
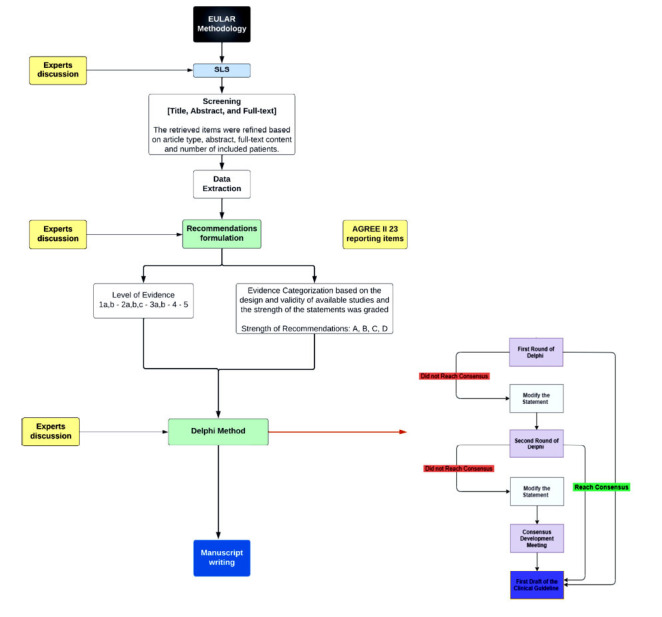
Methodology flowchart.

The Saudi group of experts (task force) has adopted the European League Against Rheumatism (EULAR) methodology to develop national clinical practice guidelines and recommendations in response to the current needs [6, 7]. Our methodology involves a rigorous and transparent process to ensure high intrinsic quality and credibility in our evidence-based recommendations (Fig. **1**). After approval from the Saudi Ministry of Health, the convenor (IH) and the methodologist (IM) have invited our multidisciplinary task force consisting of nine experts in SLE management, including rheumatologists, dermatologists, and pharmacists, to work on developing these national clinical practice recommendations. We used the Modified Delphi method and developed 31 clinical key questions covering the most important clinical aspects of SLE management, with input from all members of the Task Force. Due to the complexity and rich literature on the topic, we deferred guidelines for lupus nephritis to a separate subsequent publication. The convenor and the methodologist have worked alongside the medical writers to conduct a Systematic Literature Review (SLR) to identify relevant studies addressing the clinical key questions, following the EULAR Standardized Operating Procedures (SOPs) and the Appraisal of Guidelines Research and Evaluation instrument (AGREE II) [8]. PubMed was used to conduct the systematic literature search using the relevant search terms, and the studies were screened and selected based on our predefined inclusion and exclusion criteria. Since this guideline is adapted based on EULAR 2019 recommendations, we include all English-language publications from Jan-1995 till Nov-2022, with exceptions made for sections discussed in EULAR 2019 recommendations [7] in which the search was limited to publications after Jan-2019. More information on the search strategy can be found in Appendix **1**. The final evidence categorization, grading of recommendations, and level of agreement considered the quality of evidence that supported our recommendations/statements. The Task Force developed a consensus of 70 statements, grouped into five broad categories, and each member rated their agreement with each statement. The recommendations were formulated based on the evidence and expert consensus, considering the balance between benefits and harms, patient preferences, and resource implications. After two rounds of voting and discussion, the final statements were consolidated after getting the agreement of more than 75% of the members' votes. We ensured that our recommendations were based on the best available evidence and expert consensus and that they were relevant and applicable to clinical practice in Saudi Arabia (Supplementary Appendix **1**).

## 3 RESULTS

**Fig. (2):**
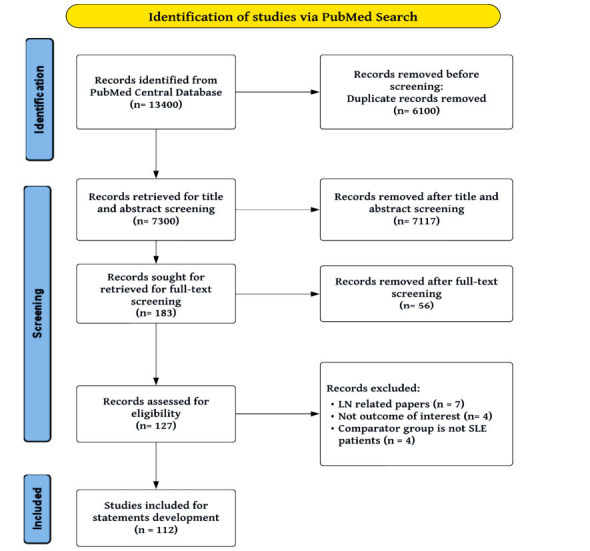
PRISMA flow chart for systematic literature review.

The search strategy retrieved 13400 records; 7300 records underwent title/abstract screening. One hundred eighty-three records were sought for retrieval, and finally, 112 studies were eligible to be included (Fig. **2**).

### 3.1 Overarching Principles

Despite the significant advances in treatment regimens that have led to a better prognosis, there are numerous challenges and unmet needs in diagnosing and treating SLE. The diagnosis of SLE is complex and requires a comprehensive evaluation of both clinical and serological findings. While classification criteria can help guide the diagnosis process, they should not be used in isolation to diagnose or exclude SLE. SLE disease activity and damage accrual monitoring are crucial in SLE management. The disease activity and damage score indices can be used to build these activities. Multidisciplinary care in lupus centers is preferable for patients with advanced disease.

### 3.2 Introduction of Common Drugs in SLE

#### 3.2.1 Glucocorticoids/Steroids

The potent anti-inflammatory properties of glucocorticoids (GCs) justify their use for managing SLE [9, 10]. GCs are used in SLE for controlling disease activity, decreasing inflammation, and preventing disease flares in SLE patients; however, several potential adverse events are related to GC usage, including osteoporosis, diabetes, hypertension, weight gain, and an increased risk of infection [11-14]. Thus, the primary goal of GC therapy in SLE patients is to establish disease control with a minimum dose and duration as well as utilize steroid-sparing agents to reduce the need for GCs [15].

The risks associated with continuous doses of GC exceeding 7.5 mg/day are significantly increased, and some studies suggest that even lower doses may be harmful. The initial dose of steroids depends on the severity of the disease and the involved organs. To optimize treatment and control disease flares, intravenous steroid pulses at varying dosages can be utilized. This approach capitalizes on the immediate non-genomic effects of GC, allowing for a lower initial dose and faster tapering [16]. In cases of acute organ-threatening disease, such as neuropsychiatric involvement, a high dose of intravenous steroids (typically 250-1000 mg/day for three days) is commonly administered, provided that infections have been ruled out [17].

The goal was to reduce the daily doses to ≤7.5 mg/day of prednisone equivalent or to stop them altogether [18, 19] to avoid long-term GC therapy-related complications, including irreversible organ damage [15, 20-22]. The ultimate goal of GC discontinuation is related to the observations of side effects, even on low doses [23]. For example, a low-dose GC, 5.0-7.5 mg prednisolone, was found to increase the infection risk, as shown in a time-dependent Cox regression analysis of 509 SLE patients from the Japanese SLE registry data suggesting that prednisolone dosage in SLE patients should be as low as possible to prevent infection [23].

A more realistic goal is Lupus Low Disease Activity State (LLDAS), which has been offered and verified as a sustainable target to be sought in SLE care [24, 25]. Less organ damage accumulation and higher quality of life have been linked to prolonged remission and LLDAS. An SLE patient with long-term minimal disease activity and no recent flare-ups for 4-6 months, even with prior severe organ involvement, is a suitable candidate to begin GC withdrawal [26-28].

#### 3.2.2 Hydroxychloroquine

All SLE patients ought to receive Hydroxychloroquine (HCQ). The advantages of HCQ in SLE are broad, and patients report improvements in constitutional symptoms, Musculoskeletal (MSK) manifestations, and mucocutaneous signs [29, 30]. Evidence from a few trials highlights that HCQ decreases the frequency of flares [30-33] and reduces thrombotic events, organ damage, and death [30, 34-36]. Despite HCQ’s various advantageous effects in SLE, medication noncompliance is prevalent [30, 37, 38]. While detecting blood drug levels is a useful compliance assessment tool and a guide to effective therapeutic level targets, its applicability in clinical practice is limited due to its unavailability in the commercial market [37, 39]. Antimalarial medications are often well tolerated, and severe adverse effects are uncommon. However, with a frequency of more than 10% for retinal abnormalities after 20 years of continuous usage, concerns about vision-threatening toxic retinopathy due to long-term HCQ treatment prompted the introduction of more sensitive screening methods [40, 41]. Baseline and regular screening is performed to detect any retinal toxicity before it causes visual impairment, and the primary screening tools are automated visual fields and Spectral Domain Optical Coherence Tomography (SD-OCT) [42]. A fundus examination of the macula should be part of the initial examination to rule out any underlying condition that might confound screening test results. Individualized risk assessment allows for flexible follow-up screening schedules throughout the first five years of therapy. The American Academy of Ophthalmology (AAO) recommends waiting five years after first exposure for follow-up tests in individuals with normal baseline exams and no significant risk factors for toxic retinopathy [42]. A daily HCQ dosage of more than 5 mg/kg actual body weight, antimalarial usage for >5 years, renal disease, concurrent tamoxifen use, and/or macular disease are all major risk factors for toxic retinopathy [42].

The daily HCQ dosage was recommended not to exceed 5 mg/kg of actual body weight (up to a maximum of 400 mg daily). While the dosing with 5 mg/kg/real BW might be associated with less eye toxicity, more recent evidence suggests a higher level might be warranted to achieve better disease activity. Several studies found a reduced flare rate associated with higher HCQ doses [43]. These findings provide preliminary evidence that reducing the daily HCQ dosage to 5.0 mg/kg/day of actual body weight may impact the frequency of flares in a population of people with SLE. Vázquez-Otero *et al.* documented that adjusting the daily dosage of HCQ to ≤5.0 mg/kg/day of real body weight did not affect the short- and mid-term outcomes [44]. There is an increased risk of SLE flares following tapering or stopping HCQs [31]; therefore, physicians should be cautious. Hyperpigmentation can occur in patients undergoing long-term treatment with chloroquine or HCQ [45]. Patients with diseases linked with easy bruising (for example, using anticoagulants or antiplatelet medications) appear to be more susceptible to developing pigmented lesions, and these lesions are typically preceded by local ecchymotic alterations [45, 46]. HCQ use is compatible with pregnancy and breastfeeding. Shared decision-making should be considered between physicians and patients who flare on doses within 5 mg/kg/real body weight regarding the use of higher doses (up to 400 mg/day, regardless of the BW) and the frequency of ophthalmological screening for toxicity.

#### 3.2.3 Immunosuppressive/Cytotoxic agents

Evidence supports using Immunosuppressive (IS) and cytotoxic agents to manage severe lupus activity affecting major organs. Consecutive administration of IS medications facilitates rapid tapering of GC and prevents disease flare-ups [47]. The agent used is determined according to the prevalent disease symptoms, the patient’s age and reproductive potential, safety considerations, and costs.

Methotrexate (MTX) demonstrated efficacy in treating joint and skin conditions, reducing disease activity, minimizing corticosteroid usage, and improving anti-dsDNA and complement levels [48]. The use of Azathioprine (AZA) has been associated with lower mortality, reduced flare frequency, and decreased CS usage in individuals with severe organ involvement [49, 50]. Its inferiority to Cyclophosphamide (CYC) led to a decline in its usage for induction in lupus nephritis throughout the subsequent decades [51, 52]. AZA is commonly used to treat extrarenal lupus and is considered a corticosteroid-sparing medication [53]. For individuals with inadequate disease control after a trial of HCQ, it is recommended to consider MTX or AZA as they are widely used and generally safe options [47].

Mycophenolate Mofetil (MMF) has been shown to be effective in renal and extra-renal manifestations [54-56]. Enteric-Coated Mycophenolate Sodium (EC-MPS) has been used in cases intolerant to MMF in many immunosuppressant regimens to improve quality of life [57]. It is not universally recommended in women of reproductive age due to its higher cost and teratogenic risk; it must be stopped at least six weeks prior to conception [58, 59]. A Cochrane systematic review echoed this finding but with poor certainty evidence [60]. Given the concerns about the reproductive toxicity of MMF and the acknowledgment that CYC has more serious reproductive risks, an alternative drug that is commonly considered for patients with organ injury in SLE is AZA. AZA is often chosen in cases where MMF might not be universally recommended, especially in women of reproductive age [61]. While AZA also has considerations and should be used cautiously, it is sometimes preferred due to its established safety profile during pregnancy compared to MMF [61]. Additionally, it does not carry the same level of reproductive toxicity as Cyclophosphamide [62, 63].

Although CYC therapy effectively treats severe organ involvement, it has been associated with teratogenicity [64, 65], hemorrhagic cystitis [66], male and female gonadal toxicity [67-74], bladder and other cancers [75-77], leukopenia [78], hyponatremia, as well as infections. During pregnancy, CYC should be avoided except for disease complications that pose grave health threats to the mother. In organ- or life-threatening occasions, administering CYC to pregnant women is an option after a detailed discussion of such treatment's potential risks and benefits with the patient or their substitute decision-maker [64, 65, 79]. Moreover, CYC therapy has been associated with lower testosterone and sperm abnormalities [66, 67]. Thus, CYC should be utilized cautiously in fertile women and men. Some evidence supports that in premenopausal individuals with SLE, utilizing Gonadotropin-Releasing Hormone agonists (GnRHa) 10-14 days before the administration of CYC therapy may reduce the risk of ovarian reserve loss [68-70]. A recent meta-analysis documented that women of reproductive age with autoimmune rheumatic disease may benefit from GnRHa when combined with intravenous CYC [71].

Calcineurin Inhibitors (CNIs) are a class of immunosuppressive agents that decrease T-cell activation by targeting the calcium/calmodulin-dependent phosphatase calcineurin [80]. Agents such as cyclosporine A and tacrolimus are widely utilized in organ transplantation. These agents decrease calcineurin activity by binding to cyclophilin and FKBP12, respectively [81]. This binding inhibits the nuclear translocation of critical transcription factors such as NF-AT, inhibiting IL-2 gene transcription [82]. Several prospective and retrospective studies have highlighted the efficacy of CNIs in addressing both renal and extrarenal manifestations of SLE [83-86]. Recent Randomized Controlled Trials (RCTs) further validate the noninferiority of CNIs compared to traditional IS agents in the induction and maintenance therapy of lupus nephritis [87-89]. Moreover, a combination of low-dose tacrolimus and MMF showed superiority over CYC pulses in inducing remission of lupus nephritis in Chinese patients [90].

Previous studies support the use of CNIs in SLE treatment, with a study by Mok *et al.* indicating significant improvement in renal function and reduced disease activity in lupus nephritis patients treated with tacrolimus [91]. Additionally, another study by Liu *et al.* highlighted the efficacy of cyclosporine in achieving remission in lupus nephritis cases [90]. These findings highlight the valuable role of CNIs as a therapeutic option for SLE, particularly in lupus nephritis.

#### 3.2.4 Biological Agents

Belimumab (BEL), a monoclonal antibody, blocks soluble human B lymphocyte stimulator from binding to B cell receptors, reducing B lymphocyte survival [72, 73], and it was the first biological therapy approved in Europe and the United States to be used for SLE. Current evidence supports BEL’s consistent efficacy against MSK and skin manifestation and satisfactory safety profile in SLE patients [74-78, 92, 93]. Add-on therapy with BEL might be considered for patients who do not adequately respond to, or are intolerant of, standard-of-care, as characterized by persistent disease activity that does not permit tapering of GC and/or recurrent relapses [7].

Anifrolumab, a monoclonal antibody to the type I interferon (IFN) receptor, was approved for treating moderate to severe SLE patients receiving standard therapy (excluding severe active lupus nephritis or neuropsychiatric SLE) [94]. The approval was based on the findings from three randomized trials demonstrating that there was a reduction in overall disease activity and GC dose with adding anifrolumab to standard therapy in the patient group compared with placebo [95-97]. In a recent study, 36.4% of patients treated with anifrolumab were GC-free (0 mg/day) at year 4, and 74.4% were receiving a dosage of 0 to 5 mg/day, which may have contributed to the decreased serious adverse events rate [98].

Anifrolumab shows a good safety profile [97]. Anifrolumab’s role in therapy is still being determined, but it appears to be especially beneficial for patients with skin and joint involvement [98] as well as hematological manifestation [98]. Anifrolumab has emerged as a potential treatment option for refractory cutaneous lupus [96, 99, 100].

The role of RTX, a B cell-depleting chimeric monoclonal antibody, in treating SLE patients remains uncertain [101]. RTX has been shown to be effective in treating SLE patients with and without lupus nephritis who have not responded to standard therapy [102-106]. Short-term improvements were seen among patients with active SLE refractory to GCs and/or immunosuppressive agents regarding disease activity, immunologic parameters, arthritis, and thrombocytopenia, as well as a GC-sparing effect [107]. On the other hand, the EXPLORER and LUNAR trials revealed no significant advantage of RTX compared with controls [108, 109]. Each study failed to assess the effectiveness of RTX because both control groups were given high dosages of GCs in addition to immune suppression. RTX is only used off-label for individuals with severe renal or extrarenal disease refractory to other IS agents or in patients with contraindications to these drugs due to the negative results of RCTs [7].

Combined therapy with RTX and BLM halted the repopulation of all B cells (including DN B cells) and simultaneously reduced SLE-relevant autoantibodies. Further research on RTX and BLM is warranted in light of the positive immunological and clinical effects shown in a cohort of patients with severe therapy-refractory SLE [110].

### 3.3 SLE Flares

SLE patients may have unexpected disease flares and remissions throughout their clinical course. While there is no universal agreement on what defines a “flare,” many physicians agree that it is an increase in the disease activity that is significant enough to warrant a therapy change [111-113]. As disease flares are prevalent and contribute considerably to the accrual of organ damage and less favorable outcomes [114-117], avoiding such flares is a crucial goal of SLE therapy [115-117]. Younger age at disease onset, non-compliance with the antimalarials, chronic generalized disease activity, and serological activity have all been documented constantly as risk factors for an increased disease flare rate [25, 118-121]. Flares can be avoided in this population by assessing adherence to pharmacological therapy, careful monitoring, and optimizing disease care. Many studies showed that belimumab and anifrolumab reduced the flare rate of SLE [122-124].

### 3.4 Gaps and Challenges Regarding the Pharmacologic Treatment of SLE in KSA

Depression and non-adherence to treatment are common among Saudi patients with SLE [125]. The complexity and duration of treatment plans might put patients at risk for failing to adhere to their medications. Medication non-adherence among SLE patients is associated with disease flares, poor quality of life, and higher expenditures to the healthcare system [126, 127]. Several factors can influence patients' compliance with medication, including access to healthcare systems and services [125, 128]. Saudi Arabia’s healthcare system offers free services to all Saudi nationals, with the government covering the costs [129]. This feature eliminates the influence of patients’ income on compliance. Despite this, up to 62.1% of the patients acknowledged medication non-adherence [125]; this is crucial for developing a strategy to increase drug adherence.

The time of diagnosis and treatment of SLE are crucial and impact survival and quality of life, as delays in therapy have been associated with a worse prognosis [130-133]. In Saudi Arabia, many patients experienced delays in accessing rheumatologists, highlighting the importance of early referral to SLE specialists or rheumatologists for accurate diagnosis and prompt treatment [134].

The agreed recommendations/statements on the pharmacologic treatment of SLE are presented in Table **1**.

### 3.5 Specific Manifestations of SLE

#### 3.5.1 Skin Involvement

The subtype of the disease influences the approach to treating skin-specific manifestations of LE. General treatment measures include stopping smoking and sun protection measures by applying 50 or greater sun protection factor sunscreen in adequate amounts (2 mg/cm^2^) at least 20 to 30 minutes before known exposure and optimizing vitamin D levels [135].

Initial therapy for cutaneous lupus erythematosus (CLE) is based on the extent and level of skin involvement. The initial approach involves topical CSs and CNIs, alongside antimalarial medications [136]. Local treatment is generally adequate for patients with Discoid lupus erythematosus (DLE) or subacute cutaneous lupus erythematosus (SCLE), which affects a small portion of the body.

Because topical CNIs are higher in cost than other topical GCs, topical GCs are frequently used as a first-line therapy [136]. Additionally, compared to topical tacrolimus, some research indicates ultra-high-potency topical GCs are more effective for DLE. The effectiveness of topical GC has been shown in several early studies [137-139]. Nevertheless, physicians and patients must be aware of the potential adverse effects of long-term use of these medications, including skin shrinkage, telangiectasia, striae, solar purpura, and hypertrichosis. Topical CNIs are preferably used on the face and other regions of delicate skin or on skin damaged by prolonged use of topical GC [140, 141].

Systemic GC therapy is an option for those with aggressive, rapidly progressive diseases. Systemic antimalarial medication is preferable when local treatment is infeasible due to widespread disease or when limited disease does not respond sufficiently to local treatment [142, 143]. Hydroxychloroquine is another standard treatment for cutaneous LE because of its efficacy and better side effect profile compared to chloroquine [144, 145]. In individuals with aggressive, rapidly progressing disease, a combination of systemic and local treatment is the most effective approach. Particularly during the initial weeks of treatment, topical GC serves as a bridge therapy until the slower-acting systemic medicines take effect. Intralesional GC may treat individuals with persistent DLE lesions that have not responded to systemic or topical CNIs [146].

MTX has demonstrated effectiveness in managing patients with refractory localized DLE with an acceptable safety profile [147]. However, certain well-known adverse effects (gastrointestinal responses, increase of liver enzymes, and pancytopenia) necessitate monitoring blood tests. AZA and CYC should not be used for CLE without systemic involvement since there is insufficient evidence in the literature and no control studies to back up their routine usage [142, 143, 148]. However, AZA, particularly in resistant leukocytoclastic vasculitis, has demonstrated good outcomes in non-specific cutaneous LE symptoms [146]. Recent studies on the use of thalidomide and lenalidomide in individuals with CLE have shown encouraging benefits; however, side effects, such as peripheral neuropathy and thromboembolic events associated with thalidomide, and cytopenia associated with lenalidomide, may restrict their usage [149]. It should only be considered a “rescue” therapy as a result of its explicit contraindication during pregnancy and the side effects [150, 151] (Supplementary Tables **1**-**3**).

Biological treatment options include belimumab and anifrolumab, which have demonstrated efficacy in treating mucocutaneous manifestations of SLE without any notable increase in significant side effects [74, 95, 99, 152-155].

The agreed recommendations/statements on the management of skin involvement in SLE are presented in Table **2**.

#### 3.5.2 Musculoskeletal Manifestations of SLE

MSK involvement is a common feature in patients with SLE. Previous studies have shown that more than 50% of Saudi SLE patients experience MSK manifestations during their disease journey, which include polyarthritis, arthralgia, and myalgia [156-159]. In addition, these symptoms can significantly impact patients' quality of life and overall disease prognosis [160].

HCQ and MTX are commonly used to manage MSK manifestations in patients with SLE. However, in cases where there is no adequate response to these medications, alternative treatment options can be considered. Biological agents such as belimumab inhibit the B-lymphocyte stimulator. Belimumab has shown promising results in improving MSK outcomes and improving the overall disease activity of SLE when other therapies have been ineffective [161-164]. Furthermore, studies on anifrolumab have demonstrated significant improvement in MSK manifestations compared to placebo. Anifrolumab also exhibits a steroid-sparing effect, which can benefit patients with SLE who require long-term CS use [97].

#### 3.5.3 Hematological Manifestations of SLE

Frequent hematological abnormalities are identified in patients with SLE, with anemia and leukopenia being the most common manifestations among Saudi SLE patients [157, 159, 165]. Anemia in SLE can result from hemolytic anemia or anemia of chronic disease and other causes (*e.g.*, bleeding), which can contribute to fatigue and diminished quality of life [157, 165, 166]. Other hematologic manifestations of SLE include thrombocytopenia and lymphopenia [4, 165-167]. Thrombocytopenia may increase bleeding risk, while lymphopenia may impair the immune system and increase susceptibility to infections [168, 169].

The treatment of such abnormalities necessitates a collaborative, multidisciplinary approach. Excluding drug-induced cytopenias is imperative in the assessment of cytopenic conditions. Mild cytopenias usually necessitate no specific treatment. However, for moderate to severe cytopenias, GCs serve as the primary therapeutic approach, with AZA, or infrequently cyclosporine-A, employed as steroid-sparing agents. In cases of severe and refractory cytopenias, a range of interventions may be considered, including IV pulse dose steroids, MMF, rituximab, CYC, plasmapheresis, recombinant Granulocyte colony-stimulating factor (G-CSF), or splenectomy [170, 171]. Additionally, Anifirulimab was used as an emerging agent to treat hematological manifestation [172]. In managing hematological manifestations within the context of SLE, a personalized approach based on the unique manifestations and individual patient characteristics is essential. Regular monitoring and collaboration among various specialists are pivotal components of optimizing patient care in these complex cases [171].

### 3.6 Treatment of Specific Conditions

#### 3.6.1 Pregnancy

Active SLE at conception is associated with a higher risk of maternal and obstetric adverse outcomes [150, 173]. Distinguishing pregnancy-related physiologic changes from disease-related symptoms during flare-ups in SLE pregnancies is challenging. Thus, optimizing maternal and fetal outcomes necessitates a multidisciplinary team, including intensive medical, obstetric, and neonatal monitoring [151]. Poor obstetric outcomes and flares should be tracked with the help of assessments of disease activity, such as renal function measures and serological markers [70]. Active disease, antihypertensive medication, earlier lupus nephritis, preeclampsia, eclampsia, antiphospholipid antibodies (aPL) presence, primigravidas, and thrombocytopenia have all been reported as indicators of unfavorable outcomes among pregnant women with SLE [174-176].

Pregnancy should be sought after six months of disease remission on pregnancy-compatible medications [177-179]. Even in the absence of active disease, medication adjustments may be required to ensure the well-being of both the mother and the fetus. A preconception evaluation is recommended for women with SLE to assess potential risks to both the fetus and mother during pregnancy.

HCQ should be continued for all SLE women during pregnancy to lower the probability of SLE flares. Patients who continue HCQ during pregnancy have shown benefits, including a lower incidence of preeclampsia, as demonstrated in previous studies [180-187].

Blood pressure monitoring, disease activity control using safe medications, especially HCQ, and limiting GC exposure are recommended in SLE pregnant patients [70, 188].

Patients with SLE have a 16-30% higher likelihood of developing preeclampsia. It has been established that initiating aspirin treatment between 12 and 16 weeks of pregnancy reduces the absolute risk of preeclampsia in women at increased risk, including SLE [189, 190]. It has been shown that combination treatment with LDA and low molecular weight heparin is more effective than monotherapy in reducing the likelihood of poor pregnancy outcomes among women with SLE-associated APS or primary APS.

Using NSAIDs beyond 20 weeks of pregnancy raises the risk of oligohydramnios; hence, the United States FDA advises only using the lowest dose that is effective for the shortest time between 20 and 30 weeks. Avoiding Nonsteroidal Anti-Inflammatory Drugs (NSAIDs) after 30 weeks of pregnancy is recommended to reduce the risk of premature closure of the ductus arteriosus and other shortcomings [191]. Treatments such as high-dose GC (which involves IV pulse therapy), IV immunoglobulin, and plasmapheresis (which can be employed in refractory nephrotic syndrome) may be explored for moderate to severe flares [192-195].

Neonatal Lupus (NL) is responsible for 80-95% of congenital complete heart block cases when structural abnormalities are not detected during prenatal or neonatal diagnosis [196, 197]. Thus, all pregnant lupus women should undergo preconception or early pregnancy testing for anti-Ro/SSA and anti-La/SSB antibodies [198].

GCs are commonly used during pregnancy to manage SLE flares. However, current evidence suggests that high-dose glucocorticoid (>20 mg/day) is associated with an increased risk of adverse pregnancy outcomes (APO), such as preterm birth and low birth weight [199-201]. Therefore, GCs should be administered a dose that is as low as possible throughout pregnancy to avoid the risk of APO.

Certain medications used to treat SLE, such as CYC, MMF, leflunomide, and MTX, are known for their teratogenicity and should be withdrawn before a planned pregnancy and avoided during pregnancy [202-204]. During the second or third trimester, CYC should be reserved for severe or refractory SLE manifestations.

HCQ, AZA, cyclosporine A, and tacrolimus are safe to be used during pregnancy [61, 70, 205]. AZA is compatible with controlling renal and extrarenal disease throughout pregnancy due to the lack of 6-MMP generation in the fetus [206, 207]. A retrospective study reported no significant differences in fetal outcomes between the SLE women exposed to AZA and those not exposed, with no major congenital abnormalities reported [61].

The safety of mycophenolate during pregnancy is a matter of concern, as it may increase the risk of birth defects, especially when used during the first trimester [202, 208]. For SLE patients who require mycophenolate for disease control, the decision to continue the medication during pregnancy should be made on a case-by-case basis, considering the patient’s disease activity, the potential teratogenicity, and the availability of other treatment options. Women of childbearing age taking mycophenolate should use effective contraception, and pregnancy should be avoided while on this medication. Conversely, Belimumab has limited data concerning its safety in SLE during pregnancy and should not be used; however, its preliminary data is reassuring [209].

#### 3.6.2 Antiphospholipid Antibodies (aPL) and Antiphospholipid Syndrome (APS)

Antiphospholipid syndrome (APS) is an autoimmune condition that leads to the occurrence of thrombosis and/or pregnancy morbidity in individuals with persistent positive test results for aPL [210]. Observational studies showed an elevated risk of arterial and venous thromboembolic events, non-thrombotic events (*i.e.* thrombocytopenia), and death in SLE patients [211-214]. A high-risk profile involves the presence of triple or double antibody positivity, while a low-risk aPL profile involves isolated aCL single antibody, anti-beta2GPI antibodies at low to medium titers, or transiently positive aPL [215, 216].

According to recommendations from the EULAR [217], due to the high likelihood of recurrent thrombosis, secondary thrombosis prevention using long-term anticoagulation is the fundamental therapy for individuals with APS. Nonetheless, several specialists maintain that the regular dose of warfarin is just as beneficial in this setting. All of these strategies for secondary prevention of arterial thrombosis are included in the EULAR guidelines [217], with the provision that the patient’s likelihood of bleeding and recurrent thrombosis must be considered. The agreed recommendations/statements on the treatment of specific conditions of SLE are presented in Table **3**.

### 3.7 Comorbidities and Adjunct Therapy for SLE

#### 3.7.1 Common Comorbidities in SLE

Patients with SLE are at increased risk of developing comorbidities such as Cardiovascular Disease (CVD), Hypertension (HTN), Diabetes Mellitus (DM), mood/cognitive disorders (particularly depression), thromboembolic events, osteoporosis, or osteopenia. CVD and HTN were among the most reported comorbidities in SLE patients in the literature [14, 218-220]. The risk of CVD increases in SLE patients due to inflammation, dyslipidemia, and other risk factors [221-223]. In previous studies, the prevalence of CVD among SLE patients ranged from 1.5% to 20% [157, 219, 219, 224-227]. In a case-control study on 571 German SLE patients, HTN was reported in 48% of the included patients [224]. Also, Walbi *et al.* reported a 20% prevalence of HTN among Saudi SLE patients [227].

DM has a high prevalence in SLE patients compared to non-SLE cohorts and is associated with increased morbidity and mortality [228]. The prevalence of DM in SLE patients might be higher than in the general population, with a prevalence among Saudi SLE patients ranging from 9% to 15% [227, 229]. The exact mechanism underlying this possible association between DM and SLE is not fully understood. Still, it is thought to be related to the chronic inflammation and immune dysregulation seen in SLE, which can lead to insulin resistance and impaired glucose metabolism [228]. Additionally, some treatments for SLE, such as corticosteroids, can also contribute to the development of DM [230, 231].

Mood and cognitive disorders, particularly depression, are prevalent among SLE patients and may be related to the disease itself or the use of corticosteroids [232]. The use of corticosteroids, often prescribed to manage SLE symptoms, may contribute to the development of these disorders. Corticosteroids can impact mood and cognition through modulation of neurotransmitter concentrations within the brain, affecting the hypothalamic-pituitary-adrenal axis and causing changes in brain structure and function [233].

Thromboembolic events, such as Pulmonary Embolism (PE), Deep Vein Thrombosis (DVT), and stroke are also more common in SLE patients due to hypercoagulability -secondary to chronic inflammation and sometimes overlap with APS- and other risk factors [234]. Previous studies reported a ~7% prevalence of DVT/PE in SLE patients [156, 166]. Finally, SLE patients are at increased risk of developing osteoporosis and osteopenia due to chronic inflammation, corticosteroid use, and other factors (such as female gender, menopause, and IS drugs) [235-237]. Albrecht *et al.* and Gergianaki *et al.* reported >20% prevalence of osteoporosis as well as osteoarthritis among SLE patients [218, 224].

Healthcare Providers (HCPs) need to be mindful of the comorbidities associated with SLE and conduct regular screenings to identify these conditions early and implement suitable management strategies. The screening process may include a range of assessments, such as clinical evaluations, laboratory tests, and imaging studies, depending on the specific comorbidity being screened for. HCPS should consider the individual patient's risk factors and tailor the screening approach accordingly [218]. Early detection and management of comorbidities in SLE patients can help optimize their overall health outcomes and improve their QOL.

#### 3.7.2 Cardiovascular Diseases

Patients with SLE are at high risk of developing CVD [236-238], and non-pharmacological interventions such as smoking cessation, maintaining a healthy weight, maintaining healthy dietary habits, and avoiding sedentary lifestyles should be considered for all SLE patients [239]. Regarding the pharmacological treatment options, HCQ use in SLE on CVD risk is reported to be beneficial. HCQ is also recommended in all cases, unless contraindicated, due to its potential atheroprotective role [240, 241].

CVD risk prediction tools may underestimate actual Cardiovascular Risk (CVR) in patients with SLE. SLE patients may be at higher risk of CVD than what is captured by traditional risk assessment tools such as cardiovascular risk score 2 (QRISK2) [242]. The adjusted global antiphospholipid syndrome score (aGAPSS) better predicted CVD events in SLE patients than QRISK3 [243]. Additionally, factors such as male gender, resting heart rate, history of lupus nephritis, initial SLE disease activity index 2000 (SLEDAI-2K) score, and metabolic syndrome were associated with increased CVD risk [243, 244]. Meanwhile, younger age at onset and the use of certain medications such as hydroxychloroquine and MMF were protective factors [244]. These findings highlight the need for tailored CVD risk assessment in SLE patients to ensure appropriate management and prevention strategies.

Studies investigating glucocorticoid treatment in SLE showed that a higher current dose was correlated with a higher risk of atherothrombotic events, ischemic heart disease (IHD), and/or stroke [21, 245]. Furthermore, hormonal therapy has been reported to induce SLE flares and cardiovascular or venous thromboembolic events [246]. Additionally, higher cumulative doses, higher daily doses, and ever-use prednisone exceeding 30 mg/day were associated with an increased risk of incident cardiovascular events in a more consistent manner [247, 248]. Therefore, as endorsed in EULAR 2022 recommendations [249], treatment with the lowest possible corticosteroid dose is recommended to minimize any potential cardiovascular risk.

There is a paucity of studies examining the management of HTN in patients with SLE, although HTN is a common comorbidity and a major risk factor for CVD. This is partly because SLE patients are often excluded from clinical trials on HTN management, leading to a lack of evidence-based guidelines for treating HTN in this population. Additionally, SLE patients may have unique pathophysiological mechanisms contributing to their HTN, such as renal involvement and inflammation [250], which require tailored management strategies.

According to the National Heart Center/Saudi Heart Association's recent guidelines [251], the initial treatment options for HTN encompass a range of antihypertensive medications. These options include Angiotensin-Converting Enzyme Inhibitors (ACEIs), Angiotensin Receptor Blockers (ARBs), diuretics (such as thiazide and thiazide-like agents), and long-acting dihydropyridine Calcium Channel Blockers (CCBs). Beta-blockers may be considered an initial treatment option in specific conditions, such as younger patients with hypertension accompanied by sympathetic overdrive, or in cases where compelling indications exist (such as heart failure, ischemic disease, obesity/bariatric surgery).

On the other hand, Beta-blockers and diuretics are better to be avoided in primary management of HTN in lupus due to the high risk of metabolic syndrome [252, 253]. Regular monitoring and treatment adjustments are recommended. As recommended in the European Society of Cardiology/European Society of Hypertension (ESC/ESH) 2007 guidelines, laboratory tests and imaging studies are necessary and should be performed annually to monitor for any changes in CVD risk factors [254]. Current studies suggest a cut point of SBP ≥130 mm Hg and/or DBP ≥80 mm Hg for diagnosis of HTN [251, 255]. Patients with specific conditions (such as APS) should also follow the same recommendations used in the general population for HTN management.

SLE patients with a high estimated risk of CVD and/or a high-risk aPL could also use Aspirin after carefully evaluating the risk of bleeding [217].

#### 3.7.3 Osteoporosis

Osteoporosis is a prevalent complication among SLE patients, often due to the inflammatory process of the disease itself, chronic use of glucocorticoids, or presence of epidemiological risk factors for bone loss (such as old age, abnormal vitamin D level, postmenopausal status, or ovarian dysfunction) [256-258]. These factors can adversely impact bone mass density (BMD) and increase SLE patients' risk of bone fractures. To improve bone health in SLE patients, HCPs should evaluate and manage risk factors that impact BMD, particularly the use of glucocorticoids. Certain lifestyle modifications, such as weight reduction, weight-bearing exercises, and smoking cessation, can also help improve bone health in these patients [259, 260]. Vitamin D and calcium supplementation have been found to significantly improve BMD, particularly in vitamin D-deficient patients [261]. A study by Caetano *et al.* found that around 50% of the postmenarcheal females with juvenile SLE had altered nutritional status. Low BMD was observed in 42.8% of patients, correlated with inadequate vitamin D supplementation [262]. Another study found that oral vitamin D and calcium supplementation in SLE patients improved BMD and reduced the frequency of osteopenia and osteoporosis without effect on SLE disease activity or related immune markers [263]. Hormone replacement therapy (HRT) can be effective for osteoporosis in patients with SLE (especially in postmenopausal females), as it can improve BMD and reduce the risk of fractures [264-267]. However, HRT use in SLE patients may also increase the risk of certain adverse effects, such as cardiovascular events, breast cancer, stroke, and venous thromboembolism [268]. Therefore, the decision to use HRT for osteoporosis in SLE patients should be carefully judged individually, considering the patient's risk factors, medical history, and personal preferences.

#### 3.7.4 Vaccination

Patients with high disease activity of systemic lupus erythematosus (SLE) benefit significantly from vaccination as they are more susceptible to infections, immune dysregulation, and frequent administration of IS therapy [269]. Vaccines can prevent infections and reduce morbidity and mortality in SLE patients [270]. However, since SLE patients have a higher risk of adverse events following vaccination, HCPs should carefully evaluate the risks and benefits of each vaccine and individualize vaccination strategies based on the medical history of the patient, disease activity, and current medication regimen.

SLE patients commonly contract viral infections during their disease journey, including Herpes Zoster (HZ), Cytomegalovirus (CMV), Human Papillomavirus (HPV), Hepatitis B and C, parvovirus B19, and influenza [269, 271]. As per our clinical practice, the most important vaccines for Saudi SLE patients include the influenza vaccine, pneumococcal vaccine, hepatitis B vaccine, and non-live HZ vaccine (SHINGRIX). Influenza is an infection that affects the respiratory system and can cause severe illness and complications in SLE patients, and the influenza vaccine is recommended for all SLE patients annually. In addition, aligned with the CDC recommendations for HZ vaccination, the Task Force members recommend using the non-live, recombinant SHINGRIX vaccine over the live attenuated Zostavax vaccine in SLE patients, even if they received IS or biologics [272].

Our recommendations align with the recent American College of Rheumatology (ACR) guidelines on vaccination [273]. The pneumococcal vaccine is also recommended for SLE patients, as they are at increased risk of pneumococcal infections, which can lead to pneumonia, meningitis, and sepsis [274]. Also, the hepatitis B vaccine is suggested for SLE patients due to the increased risk of hepatitis B infection, which can cause liver damage and other complications [275]. Additionally, due to lifelong IS drug use, patients with SLE are at higher risk of HPV infection and its possible complications [276-278]. Therefore, HPV vaccines should be considered for SLE patients, particularly those with a history of cervical dysplasia or human papillomavirus infection. The agreed recommendations/statements on the common comorbidities and adjunct therapy for SLE are presented in Table **4**.

We have compiled Table **5** to illustrate the differences and deviations between our recommendations and EULAR guidelines.

## CONCLUSION

Systemic Lupus Erythematosus (SLE) has a high prevalence in Saudi Arabia, causing a burden on both patients and healthcare systems. The disease and many confounding factors play a role in determining the most suitable treatment plan for each patient.

GCs are the cornerstone treatment that helps control disease and prevent flares. However, they cause many unwanted adverse events, including hypertension and diabetes. Hydroxychloroquine is also used as SLE treatment, leading to significant improvements in symptoms as well as a decrease in the flare frequencies. Cytotoxic and immunosuppressive agents could manage the severe organ damage caused by lupus. Monoclonal antibodies also play a significant role in managing SLE.

A good treatment plan should be carefully customized for each patient to decrease the number of flares during the clinical course of the disease and to encourage the patients to adhere to medications.

Many comorbidities, including cardiovascular diseases, mood/cognitive disorders, and osteoporosis, affect SLE patients and could endanger their safety. Therefore, monitoring plans, adjunct therapy, and vaccines should be implemented for them.

## RECOMMENDATIONS FOR FUTURE RESEARCH

Future research is required to develop precision medicine approaches in SLE based on genetic, environmental, and clinical factors to enhance diagnosis and treatment. It is also necessary to explore the development of new medications, addressing unmet needs such as long-term remission maintenance and steroid-sparing agents. Furthermore, long-term clinical trials are required to assess the safety and efficacy of current and emerging therapies for SLE, focusing on high-risk populations such as children, patients with comorbidities, and pregnant women. We should aim to identify better diagnostic tools and biomarkers for accurate SLE diagnosis and monitoring of disease activity over time. Studies into SLE within the Saudi population are necessary to comprehend disease prevalence, risk factors, clinical manifestations, and treatment outcomes specific to this demographic.

## AUTHORS’ CONTRIBUTIONS

Each author listed has significantly contributed to the work, providing substantial, direct, and intellectual input, and has given their approval for its publication.

## Figures and Tables

**Table 1:** Expert recommendations and statements on pharmacologic treatment of SLE.

**Statement/Recommendation**	**Percentage of Agreement**
**Glucocorticoids/Steroids**	
Whenever a high dose of steroids is needed, we recommend pulses of IV methylprednisolone (typically 250-1000 mg daily, for 1-3 days) in order to achieve an immediate therapeutic effect while minimizing steroid exposure through the use of a reduced initial dose of oral GC.	100%
GC dosage for chronic maintenance regimens should be decreased to less than 5 mg daily (prednisone equivalent) and, when feasible, discontinued.	100%
Withdrawing GC medications is possible after achieving remission or a lupus low disease activity state (LLDAS). This can help reduce the risk of side effects associated with long-term GC use. However, careful monitoring is crucial as flares can still occur after stopping GC.	77%
Despite the benefits supporting GC withdrawal, close observation after withdrawal is required and advisable due to the risk of a flare-up.	89%
Rapid initiation of immunomodulatory drugs can hasten the GC tapering/discontinuation.	89%
**Hydroxychloroquine**	
Previously, the maximum dose of HCQ was 5 mg/kg/real BW, while recent evidence supports higher dosing (up to 400 mg/day, regardless of the BW).	100%
The current evidence regarding the impact of HCQ dose tapering on the short-term and midterm outcomes in SLE is controversial. Therefore, the decision to maintain or reduce HCQ should be personalized according to different subgroups of patients.	88%
In patients who are using an HCQ dose higher than 5 mg/kg/real BW or have renal impairment, ophthalmological screening (by visual fields examination and/or spectral domain-optical coherence tomography) should be performed on an annual basis.	77%
**Immunosuppressive/Cytotoxic Agents**	
The choice of immunosuppressive/cytotoxic agents depends on the predominant disease symptom(s), age, possibility of pregnancy, and safety considerations.	100%
MTX and AZA should be taken into consideration for patients who have poor symptom control following a trial with GC and HCQ or when HCQ alone is not sufficient due to the experience acquired with their utilization and their overall safe profile.	100%
In organ-threatening diseases (especially renal, cardiopulmonary, or neuropsychiatric) CYC may be used and only as a rescue therapy for refractory non-major organ symptoms; due to its gonadotoxic effects, it should be used with caution in patients of reproductive age.	100%
Mycophenolate mofetil (MMF) or enteric-coated mycophenolate sodium can be used instead of CYC and or azathioprine (AZA) in patients with active systemic lupus erythematosus.	89%
**Biological Agents**	
For patients who do not respond adequately to standard-of-care treatments (combinations of HCQ, GC, and immunosuppressive agents) or who develop intolerance to them (as defined by residual disease activity that prevents tapering of glucocorticoids and/or frequent relapses), additional treatment with belimumab and anifrolumab should be contemplated.	100%
Belimumab or anifrolumab are considered therapeutic options for patients with SLE with mucocutaneous and/or musculoskeletal manifestations with a manageable safety profile.	100%
The only indications for off-label prescription of RTX are in patients who have severe renal or extrarenal (mainly hematological and neuropsychiatric) disease resistant to other IS agents and/or belimumab and anifrolumab or when these drugs are contraindicated.	100%
**SLE Flares**	
Preventing the flares represents an extra milestone of SLE treatment. Although there is no agreed-upon definition, the majority of experts agree that a flare is a measurable escalation in the activity of a disease, usually leading to treatment change.	100%
Assessment of adherence to drug treatment, close monitoring, and optimization of disease control in these patients may reduce the risk of a flare.	100%
GC withdrawal should be made with caution, especially in patients with serologically active yet clinically quiescent to avoid flare.	100%
**Gaps and Challenges**	
Medication adherence is not optimal in SLE patients, including Saudi populations. A routine review of treatment compliance during each visit may ensure medication adherence.	100%

**Table 2:** Expert recommendations and statements on the management of skin involvement in SLE.

**Statement/Recommendation**	**Percentage of Agreement**
**Skin Involvement**	
For patients with Cutaneous Lupus Erythematosus (CLE), general measures to be considered include smoking cessation and sun protection measures by applying 50 or greater Sun Protection Factor (SPF) sunscreen in adequate amounts (2 mg/cm^2^) at least 20 to 30 minutes before known exposure in addition to optimization of vitamin D levels.	100%
Topical agents (GC and/or CNIs) and antimalarials, with or without systemic GC, depending on the severity of skin involvement, are the recommended first-line treatment for SLE.	100%
Prolonged use of topical corticosteroids is known to cause atrophy, telangiectasia, and steroid-induced rosacea-like dermatitis. Therefore, topical CNIs can be used as effective steroid-sparing agents in areas at high risk of steroid complications (*e.g.*, facial skin).	100%
HCQ is preferred as antimalarial over chloroquine due to its numerous beneficial effects and feasibly lower risk for retinal toxicity.	100%
MTX or other agents, such as retinoids, dapsone, MMF, or EC-MPS, can be used when first-line treatment fails to show a response in SLE.	100%
Belimumab and anifrolumab can be considered in resistant mucocutaneous manifestations of SLE after the failure of immunosuppressive therapy.	100%

**Table 3:** Expert recommendations and statements on the treatment of specific conditions and monitoring of SLE.

**Statement/Recommendation**	**Percentage of Agreement**
**Pregnancy**	
Current evidence shows maternal and fetal complications were significantly higher in SLE-associated pregnancies. Therefore, SLE should be considered a high risk for pregnancy.	89%
Given the possible complications and morbidity, pregnant women with SLE are regarded as high risk for adverse outcomes of pregnancy and should be under the care of a multidisciplinary team, which ideally consists of a rheumatologist, an obstetrician with lupus expertise, an internist, and, if necessary a nephrologist.	100%
The status of SLE is intimately correlated with maternal and neonatal outcomes. Thus, accurate prediction of at-risk females before conception is crucial to avoid the negative impact of SLE on pregnancy outcomes.	100%
Prior to attempting pregnancy, remission or low lupus disease activity state (LLDAS) is the desired condition before pregnancy. Good pregnancy outcomes could be achieved in case of remission and adequately controlled disease activity before pregnancy.	100%
Counseling prior to conception is recommended for women with SLE and/or APS to implement preventive strategies and develop a personalized plan for monitoring before and during pregnancy.	100%
Patients should be in LLDAS or remission for 4-6 months prior to attempting conception on pregnancy-compatible medications. Pregnancy should be postponed for individuals with moderate to severe disease activity until the condition is effectively managed with stable, pregnancy-compatible medications.	100%
HCQ is recommended prior to conception and during pregnancy for patients with SLE.	100%
HCQ can improve pregnancy outcomes in SLE patients by reducing the risk of preeclampsia. All patients should use HCQ as long as it is not contraindicated.	78%
Blood pressure monitoring, using safe medications to control disease activity, especially HCQ, and limiting glucocorticoid exposure are essential measures.	100%
In pregnant women with SLE, assessment of disease activity, including renal function parameters and serological markers (serum C3/C4, anti-dsDNA titers), is recommended to monitor adverse obstetrical outcomes and disease flares.	100%
LDA should be administrated to women with SLE at risk of preeclampsia (particularly those with lupus nephritis or positive aPL). In women with SLE-associated APS or primary APS, combination therapy with LDA and heparin is suggested to decrease the risk of pregnancy adverse outcomes.	89%
Current evidence shows that Non-Steroidal Anti-Inflammatory Drugs (NSAIDs) can be used during the first and second trimesters.	100%
Additional therapeutic approaches, such as glucocorticoids, intravenous pulse therapy, intravenous immunoglobulin, and plasmapheresis, can be used to manage moderate to severe flares.	89%
Current evidence shows an increased risk of pregnancy adverse outcomes with glucocorticoid use (> 20 mg/day of prednisolone), especially preterm birth and low birth weight. Prednisolone/prednisone can be used throughout pregnancy at the lowest effective dose.	100%
Mycophenolic acid, CYC, leflunomide, and methotrexate should be avoided during pregnancy due to known or possible teratogenicity.	100%
Current evidence shows that CYC is associated with a higher incidence of congenital malformations. Withdrawal of CYC is required prior to a planned pregnancy.	100%
CYC should not be used during the first trimester of pregnancy due to the risk of fetal loss. It should be reserved exclusively for the treatment of severe, life-threatening, or refractory SLE manifestations throughout the second or third trimester.	100%
Current evidence indicates an increased rate of congenital malformation with methotrexate. In a planned pregnancy, withdrawal of methotrexate should be three months prior to pregnancy.	89%
HCQ, oral glucocorticoids, azathioprine, ciclosporin A, and tacrolimus can be used to prevent or manage SLE flares during pregnancy.	100%
Patients who showed a response to initial treatment with MPA should continue to take it, but once the pregnancy is planned, a pregnancy- planned transition to AZA is recommended at least six weeks prior to conception.	100%
Mycophenolate should be discontinued six weeks prior to conception. The teratogenic potential of enteric-coated mycophenolate sodium prevents it from being recommended universally for women of reproductive age who have non-renal manifestations.	89%
Preliminary data about the safety of Belimumab during pregnancy is reassuring; nevertheless, more data are needed. Using such agents in pregnant SLE patients should be approached with caution and close monitoring.	89%
Thalidomide exhibits efficacy in a range of subtypes of cutaneous disorders. On account of its explicit contraindication during pregnancy, the risk for irreversible polyneuropathy, and the frequent relapses associated with discontinuing the drug, its use should be limited to patients who have tried and failed numerous prior therapeutic agents as a “rescue” measure.	100%
**Antiphospholipid Antibodies (aPL) and Antiphospholipid Syndrome (APS)**	
All patients with SLE should be screened at diagnosis for aPL due to the high risk of thrombotic events, adverse fetal outcomes, non-thrombotic events, and mortality.	88%
After balancing the bleeding hazard, patients with SLE who have a profile of elevated risk for aPL (persistently positive medium/high titers or multiple positivity) may be offered primary prophylaxis with ASA, particularly in the presence of other atherosclerotic/thrombophilic factors.	100%
The therapeutic strategy for secondary prevention (thrombosis, pregnancy complications/loss) should be identical to that used for primary antiphospholipid syndrome.	77%
**Monitoring and Optimal Treatment Targets**	
aPL, Anti-Ro, and anti-La antibodies should be checked prior to pregnancy.	78%
Comorbidities, including atherosclerotic disease, avascular necrosis, osteoporosis, malignancy, and infection, are more prevalent among patients with lupus. It is important to review the management of modifiable risk factors, including diabetes, hypertension, dyslipidemia, high BMI, and smoking.	100%
Immunosuppressive therapy and hydroxychloroquine both have the potential to induce toxicities. Drugs should be closely monitored by routine laboratory tests, and clinical evaluations should be done following the guidelines for drug monitoring.	100%
If the remission is unachievable, SLE Treatment should aim to decrease disease activity in all organ systems.	100%
Data concerning the optimal timing and duration of therapy discontinuation in renal and extrarenal disease are scarce. It is possible to attempt a gradual withdrawal of immunosuppressive agents following a complete clinical response for a minimum of three to five years of therapy. Hydroxychloroquine should be continued long-term.	100%

**Table 4:** Expert recommendations and statements on comorbidities and adjunct therapy.

**Recommendation/Statement**	**Percentage of Agreement**
**The Most Common Comorbidities in SLE**	
The high prevalence of multimorbidity among patients with SLE in the community advocates for multidisciplinary care to optimize clinical outcomes.	89%
**Comorbidities Screening**	
Screening for various comorbidities at SLE diagnosis is recommended to reduce organ damage, complications, and mortality risk.	100%
Patients with SLE should adhere to the screening recommendations for the general population, particularly for cervical cancer and cardiovascular diseases.	100%
**Cardiovascular Diseases**	
Clinicians need to be aware of the increased cardiovascular risk among patients with SLE. Therefore, non-pharmacological interventions for CVD, such as smoking cessation, avoiding sedentary lifestyles, and maintaining an optimal BMI, should be considered for all SLE patients.	100%
Unless contraindicated on account of its hypothesized atheroprotective effect, HCQ could be used in all lupus cases.	89%
Hormone replacement therapy (HRT) is better avoided in SLE patients due to the associated increased risk of CVD and venous thromboembolism.	89%
ACEIs or ARBs (in case of intolerance) are preferred as first-line treatment for HTN in patients with SLE due to their renoprotective effects (*i.e.*, improve serum creatinine levels and reduce proteinuria).	100%
If the blood pressure cannot be controlled by monotherapy alone or when concurrent pulmonary arterial hypertension is present, a calcium channel blocker (CCB) or thiazide diuretic should be added.	78%
All SLE patients who have begun antihypertensive medication are recommended to return at least every two months for monitoring and treatment adjustment until they achieve their BP goal.	78%
**Osteoporosis**	
Factors adversely impacting BMD, particularly chronic use of glucocorticoids, should be evaluated and managed.	100%
To improve bone health in SLE patients, certain lifestyle changes such as weight loss, weight-bearing exercises, and smoking cessation should be implemented.	100%
Vitamin D and calcium supplementation significantly improved the BMD in vitamin D-deficient SLE patients.	100%
Due to the high prevalence of osteoporosis and osteopenia among Saudi SLE patients, screening for BMD is advocated, especially in high-risk patients (such as the elderly and patients on chronic GC therapy).	100%
**Vaccination**	
Adult patients with SLE should be urged to receive vaccinations according to Saudi national guidelines. In addition, influenza, pneumococcal vaccination, and SHINGRIX (Zoster Vaccine Recombinant, Adjuvanted) should be considered in all SLE patients, irrespective of their treatments.	100%

**Table 5:** Changes/Deviations between our guidelines and EULAR recommendations.

**Statement/Recommendation**
**Glucocorticoids/Steroids**
EULAR guidelines may recommend standard oral glucocorticoid regimens for high-dose needs, while our recommendation emphasizes pulses of IV methylprednisolone.
While EULAR guidelines suggest a tapering approach for glucocorticoid maintenance, we recommend decreasing chronic regimens to less than 5 mg daily.
**Hydroxychloroquine**
EULAR guidelines might propose a maximum dose of HCQ based on body weight, whereas we support higher doses (up to 400 mg/day) regardless of weight.
While EULAR may suggest a standardized approach to HCQ maintenance, our recommendation is for personalized decisions on maintenance or reduction based on patient subgroups.
**Immunosuppressive/Cytotoxic Agents**
EULAR guidelines may prioritize certain immunosuppressive agents in specific scenarios, but our recommendation considers MTX and AZA earlier in the treatment sequence.
EULAR guidelines may have a more prominent role for CYC in certain cases, but our recommendation limits its use and favors MMF or enteric-coated mycophenolate sodium.
**Biological Agents**
EULAR guidelines might have specific criteria for considering belimumab or anifrolumab, but our recommendation includes these as therapeutic options for patients not responding adequately or developing intolerance.
The off-label use of RTX in specific cases may deviate from EULAR guidelines, which might have different recommendations for severe renal or extrarenal disease.
**Skin Involvement**
EULAR guidelines may provide recommendations on first-line treatments for cutaneous lupus erythematosus (CLE), but our recommendation emphasizes certain measures like smoking cessation and sun protection.
Our recommendation includes specific alternatives such as belimumab and anifrolumab for resistant mucocutaneous manifestations, which might differ from EULAR guidelines.
**SLE Flares**
While EULAR guidelines may have a definition for flares, our recommendation provides a measurable escalation in disease activity.
Our emphasis on assessing adherence and close monitoring may go beyond the general recommendations in EULAR guidelines.
Gaps and challenges
The EULAR guidelines may not explicitly address medication adherence issues in Saudi populations, whereas our recommendation highlights this as a significant gap.
**Pregnancy**
EULAR may recommend standardized oral glucocorticoid regimens during pregnancy, while our recommendation emphasizes the use of HCQ as a preventive measure.
EULAR may recommend standardized oral glucocorticoid regimens during pregnancy, while our recommendation emphasizes the use of HCQ as a preventive measure.
The use of NSAIDs during the first and second trimesters is supported in our recommendation, while EULAR guidelines may have different considerations.
EULAR guidelines may provide more specific recommendations for the use of glucocorticoids during pregnancy, whereas our recommendation suggests prednisolone/prednisone at the lowest effective dose.
Our recommendation emphasizes a cautious approach to CYC, particularly avoiding its use during the first trimester, which may differ from EULAR guidelines.
The recommendation to transition from MPA to AZA six weeks before conception may deviate from EULAR guidelines.
The consideration of Belimumab during pregnancy is approached with caution and close monitoring in our recommendation, which might differ from EULAR guidelines.
**Antiphospholipid Antibodies (aPL) and APS**
Our recommendation includes screening all SLE patients for aPL at diagnosis, while EULAR guidelines may have different criteria.
The primary prophylaxis with ASA in patients with elevated aPL risk factors may differ from EULAR guidelines.
Our recommendation suggests an identical therapeutic strategy for secondary prevention in patients with SLE and APS, which may not align exactly with EULAR guidelines.
**Monitoring and Optimal Treatment Targets**
Our recommendation includes checking aPL, Anti-Ro, and anti-La antibodies prior to pregnancy, which may be more specific than EULAR guidelines.
The emphasis on reviewing and managing modifiable risk factors is in line with general guidelines but may be more explicitly stated in our recommendation.
Routine laboratory tests and clinical evaluations for monitoring immunosuppressive therapy and hydroxychloroquine align with general principles but may be emphasized more explicitly in our recommendation.
The suggestion to attempt a gradual withdrawal of immunosuppressive agents after a complete clinical response for three to five years may not be specifically outlined in EULAR guidelines.
**The Most Common Comorbidities in SLE**
Both our recommendation and EULAR guidelines advocate for multidisciplinary care to optimize clinical outcomes, showing alignment in this aspect.
**Comorbidities Screening**
The recommendation to screen for various comorbidities at SLE diagnosis aligns with general principles in EULAR guidelines.
The emphasis on adhering to general population screening recommendations, particularly for cervical cancer and cardiovascular diseases, is in line with EULAR guidelines.
**Cardiovascular Diseases**
Non-pharmacological interventions for cardiovascular diseases are emphasized in both recommendations and general EULAR guidelines.
The hypothesized atheroprotective effect of HCQ is acknowledged in both recommendations.
The avoidance of hormone replacement therapy (HRT) in SLE patients due to increased CVD and venous thromboembolism risk aligns with general principles.
The preference for ACEIs or ARBs as first-line treatment for hypertension, considering their renoprotective effects, is in line with general EULAR guidelines.
The recommendation to add a calcium channel blocker (CCB) or thiazide diuretic if blood pressure control is inadequate aligns with general hypertension management principles.
The suggested monitoring and treatment adjustment every two months for SLE patients on antihypertensive medication is more specific but aligns with the general goal of achieving blood pressure control in EULAR guidelines.
**Osteoporosis**
The evaluation and management of factors adversely impacting bone mineral density (BMD), particularly chronic use of glucocorticoids, are in line with general EULAR guidelines.
Lifestyle changes such as weight loss, weight-bearing exercises, and smoking cessation to improve bone health align with general principles.
Vitamin D and calcium supplementation for improving BMD in vitamin D-deficient SLE patients is a general recommendation.
The advocacy for screening for BMD, especially in high-risk patients, aligns with general EULAR guidelines.
**Vaccination**
The urging of adult SLE patients to receive vaccinations according to Saudi national guidelines aligns with general principles.
The consideration of influenza, pneumococcal vaccination, and SHINGRIX for all SLE patients, irrespective of treatments, aligns with general vaccination recommendations.

## LIST OF ABBREVIATIONS

AAO: American Academy of Ophthalmology
ACEIs: Angiotensin-converting Enzyme Inhibitors
aCL: Anticardiolipin
aGAPSS: Adjusted Global Antiphospholipid Syndrome Score
AGREE II: Appraisal of Guidelines Research and Evaluation Instrument
APL: Antiphospholipid Antibodies
APS: Antiphospholipid Syndrome
ARBs: Angiotensin Receptor Blockers
AZA: Azathioprine
BEL: Belimumab
BICLA: British Isles Lupus Assessment Group-based Composite Lupus Assessment
BILAG: British Isles Lupus Assessment Group
BMD: Bone Mass Density
CLE: Cutaneous Lupus Erythematosus
CMV: Cytomegalovirus
CNIs: Calcineurin Inhibitors
CVR: Cardiovascular Risk
CYC: Cyclophosphamide
DLE: Discoid lupus Erythematosus
DM: Diabetes Mellitus
DVT: Deep Vein Thrombosis
EC-MPS: Enteric-coated Mycophenolate Sodium
ESC/ESH: European Society of Cardiology/European Society of Hypertension
EULAR: European League Against Rheumatism
GCs: Glucocorticoids
GnRHa: Gonadotropin-releasing Hormone Agonists
HCPs: Healthcare Providers
HCQ: Hydroxychloroquine
HPV: Human Papilloma Virus
HR: Hazard Ratio
HRT: Hormone Replacement Therapy
HTN: Hypertension
HZ: Herpes Zoster
IFN: Interferon
IHD: Ischemic Heart Disease
IS: Immunosuppressive
LA: Lupus Anticoagulant
LLDAS: Lupus Low Disease Activity State
MMF: Mycophenolate Mofetil
MSK: Musculoskeletal
MTX: Methotrexate
NL: Neonatal Lupus
NSAIDs: Nonsteroidal Anti-inflammatory Drugs
OR: Odds Ratio
PE: Pulmonary Embolism
QOL: Quality of Life
RCTs: Randomized Controlled Trials
RTX: Rituximab
SCLE: Subacute Cutaneous Lupus Erythematosus
SD-OCT: Spectral-domain Optical Coherence Tomography
SLE: Systemic Lupus Erythematosus
SLEDAI: Systemic Lupus Erythematosus Disease Activity Index
SLEDAI-2K: Systemic Lupus Erythematosus Disease Activity Index 2000
SLR: Systematic Literature Review
SOPs: Standardized Operating Procedures
SRI-4: SLE Responder Index 4
VTE: Venous Thromboembolism
