# Supplementary material for: Saudi National Clinical Practice Guidelines for Management of Adult Systemic Lupus Erythematosus
Source: Curr Rheumatol Rev. 2024 Apr 30;21(1):70–96. doi: 10.2174/0115733971275638240429063041 (PMC12079323; doi:10.2174/0115733971275638240429063041)
Supplement: Supplementary file 1 [file CRR-21-1-70_SD1.pdf]

Supplementary Material

Saudi National Clinical Practice Guidelines for Management of Adult Systemic Lupus Erythematosus

Ahmed H. Al-jedai[1,2], Hajer Y. Almudaiheem[1], Ibrahim A. Al-Homood[3,4], Ibrahim Almaghlouth[5,6], Sami M. Bahlas[7], Abdulaziz Mohammed Alolaiwi[1,8], Mohammad Fatani[9], Maysa Tariq Eshmawi[10,11], Bedor A. AlOmari[12], Khalidah Ahmed Alenzi[13], Rayan G. Albarakati[14] and Nayef Al Ghanim[15,*]

[1]Deputyship of Therapeutic Affairs, Ministry of Health, Riyadh, Saudi Arabia; [2]Colleges of Medicine and Pharmacy, Al Faisal University, Riyadh, Saudi Arabia; [3]Medical Specialties Department, King Fahad Medical City, Riyadh, Saudi Arabia; [4]Medicine Department, College of Medicine, Al Faisal University, Riyadh, Saudi Arabia; [5]Department of Medicine, College of Medicine, King Saud University. Riyadh 11461, Saudi Arabia; [6]College of Medicine Research Center, King Saud University, Riyadh 11461, Saudi Arabia; [7]Department of Internal Medicine, Faculty of Medicine, King Abdulaziz University, Jeddah 21589, Saudi Arabia; [8]Department of Rheumatology, King Saud Medical City, Riyadh, Saudi Arabia; [9]Hera General Hospital, Ministry of Health, Makkah, Saudi Arabia; [10]King Abdullah Medical Complex, Jeddah, Saudi Arabia; [11]College of Medicine, Imam Mohammad Ibn Saud Islamic University, Riyadh, Saudi Arabia; [12]Department of Pharmaceutical Services, Prince Sultan Military Medical City Riyadh, Saudi Arabia; [13]Tabuk Health Cluster, Tabuk, Saudi Arabia; [14]Department of Obstetrics and Gynecology, Majmaah University, Al-Majmaah 11952, Saudi Arabia; [15]Department of Rheumatology, King Saud Medical City, Riyadh, Saudi Arabia

Appendix 1: Search Strategy for SLE KSA Guidelines (SLS strategy)

Section	Databases search strings
**Pharmacologic treatment of SLE**	
1. What is the evidence for the benefits and harms of glucocorticoids in treating SLE?<br>2. What is the optimal starting dose and duration of glucocorticoids in treatment of SLE?<br>3. What is the optimal maintenance dose of steroids in SLE?<br>4. What is the evidence for the benefits and harms of hydroxychloroquine in treating SLE?<br>5. What is the evidence for the benefits and harms of immunosuppressive/cytotoxic agents in treating SLE?<br>6. What is the evidence for the benefits and harms of methotrexate and other antimetabolites in treating SLE?<br>7. What is the evidence for the benefits and harms of calcineurin inhibitors in treating SLE?<br>8. What is the evidence for the benefits and harms of biologics in treating SLE and relapsing SLE?	((systemic lupus erythematosus) OR SLE [Title] OR DLE[Title] OR "lupus"[Title] OR SCLE[Title] OR CLE[Title]) **AND** (glucocorticoid* OR steroid* OR corticosteroid* OR methylprednisolone OR (intravenous methylprednisolone) OR hydroxychloroquine OR antimalarial* OR quinacrine OR methotrexate OR leflunomide OR calcineurin OR cyclosporin OR tacrolimus OR voclosporin OR azathioprine OR mycophenolate OR mycophenolic OR cyclophosphamide OR rituximab OR abatacept OR belimumab OR anifrolumab OR biologic* OR (intravenous immunoglobulin) OR (plasma exchange) OR plasmapheresis)

1875-6360/25 © 2025 Bentham Science Publishers

9. What are the current gaps/status/challenges regarding the pharmacologic treatment of SLE that need to be tackled in Saudi Arabia?	((systemic lupus erythematosus) OR SLE [Title] OR DLE[Title] OR "lupus"[Title] OR SCLE[Title] OR CLE[Title]) **AND** (glucocorticoid* OR steroid* OR corticosteroid* OR hydroxychloroquine OR antimalarial* OR quinacrine OR methotrexate OR leflunomide OR calcineurin OR cyclosporin OR tacrolimus OR voclosporin OR azathioprine OR mycophenolate OR mycophenolic OR cyclophosphamide OR rituximab OR belimumab OR anifrolumab OR abatacept OR biologic* OR (intravenous immunoglobulin) OR (plasma exchange) OR plasmapheresis) **AND** ((saudi Arabia) OR saudi)
10. How should SLE flares be treated?	((systemic lupus erythematosus) OR SLE[Title] OR "lupus"[Title]) **AND** (flare*[Title] OR flaring[Title] OR exacerbat*[Title] OR relaps*[Title])
**Treatment of specific manifestations**	
11. How should skin involvement in SLE be treated?	("lupus rash"[Title] OR "malar rash"[Title] OR "skin lupus"[Title] OR "cutaneous lupus"[Title] OR DLE[Title] OR "discoid lupus"[Title] OR SCLE[Title] OR CLE[Title] OR "chilblain lupus"[Title] OR "lupus tumidus"[Title] OR "lupus panniculitis"[Title]) **AND** ("sun protection" OR "topical" OR retinoid* OR thalidomide OR lenalidomide OR dapsone)
12. How should neuropsychiatric (NP) involvement in SLE be treated?	Identical search as per questions 1 to 8 with an additional hand search of retrieved articles
13. Management of antiphospholipid syndrome in SLE	((systemic lupus erythematosus) OR SLE[Title] OR "lupus"[Title]) **AND** (anti-b2* OR anti-beta* OR anti-β2* OR anti-cardiolipin* OR anticardiolipin* OR "lupus anticoagulant" OR LAC OR aPL) **AND** (syndrome OR APS OR thrombosis OR thrombotic OR vascular OR pregnan* OR obstetric*) **AND** (management OR therapy OR therapeutic OR treatment OR anticoagul* OR antiplatelet* OR anti-platelet* OR aspirin OR heparin OR warfarin)
14. What are the current gaps/status/challenges regarding the treatment of SLE-specific manifestations that need to be tackled in Saudi Arabia?	The same as questions 10 – 13 with adding (Saudi Arabia)
**Monitoring and treatment targets**	
15. How often and by which means should disease activity and damage be assessed in SLE?	((systemic lupus erythematosus) OR SLE[Title] OR "lupus"[Title]) **AND** (treatment OR therapy OR management) **AND** (target[Title] OR aim[Title] OR response[Title] OR inactive[Title] OR remission[Title/Abstract] OR "low disease activity"[Title/Abstract])
16. What are the optimal treatment targets in SLE?	
17. What is the optimal duration of immunosuppressive/biologic treatment in SLE?<br>18. What is the optimal duration of transitioning conventional systemic therapy to biological therapy?<br>19. What is the optimal duration of transitioning from	((systemic lupus erythematosus) OR SLE [Title] OR "lupus"[Title]) **AND** (treatment OR therapy OR management) **AND** (stop*[Title/Abstract] OR withdraw*[Title/Abstract] OR discontin*[Title/Abstract] OR taper*[Title/Abstract] OR duration [Title/Abstract])

one biologic to another?	
20. What are the current gaps/status/challenges regarding the monitoring of SLE that need to be tackled in Saudi Arabia?	The same as questions 15 to 19 with adding (Saudi Arabia)
**Comorbidities and adjunct therapy for SLE**	
21. How should comorbidities be managed in SLE?	((systemic lupus erythematosus) OR SLE[Title] OR "lupus"[Title]) AND (comorbid*[Title] OR infectio*[Title] OR vaccin*[Title] OR immuniz*[Title] OR osteoporo*[Title] OR bone[Title] OR cardiovascular[Titlle] OR atherosclero*[Title] OR myocardial[Title] OR angina[Title] OR heart[Title] OR "peripheral arterial"[Title] OR "peripheral vascular"[Title] OR claudication[Title] OR hypertension[Title] OR dyslipidaemia[Title] OR hypercholesterol*[Title] OR diabetes[Title] OR hyperglycem*[Title] OR cancer[Title] OR malignan*[Title] OR fatigue[Title] OR pain[Title])
22. What are the current gaps/status/challenges regarding the management of SLE comorbidities that need to be tackled in Saudi Arabia?	The same as question 21 with adding (Saudi Arabia)

**SLE Comorbidities**<br>23. What is the definition of comorbidities in SLE?<br>24. What is the most common comorbidities in SLE?<br>25. What is the evidence of comorbidities screening in SLE?<br>26. What is evidence for comorbidities management in SLE?<br>27. What is the evidence of vaccination in SLE?<br>28. What is the evidence of frailty management in SLE?	((systemic lupus erythematosus) OR SLE [Title] OR DLE[Title] OR "lupus"[Title] OR SCLE[Title] OR CLE[Title]) **AND** (comorbid*[Title] OR thyroid[Title] OR hypertension[Title] OR dyslipidemia[Title] OR dyslipoproteinemia*[Title] OR obesity[Title] OR infection*[Title] OR osteoporosis[Title] OR bone[Title] OR cardiovascular[Title] OR cardiac[Title] OR heart[Title] OR allergic[Title] OR mental[Title] OR depression[Title] OR “generalized anxiety disorder” [Title] OR frailty[Title] OR geriatric[Title] OR vaccination[Title] OR vaccin*[Title])
**Cardiopulmonary manifestations**<br>29. What is the evidence of risk scores for CVS risk among SLE patients?<br>30. What are the current gaps/status/challenges regarding the treatment of CVS risks in SLE?<br>31. Similar questions for the rest of the damage items?	((systemic lupus erythematosus) OR SLE [Title] OR DLE[Title] OR "lupus"[Title] OR SCLE[Title] OR CLE[Title]) **AND** (cardiopulmonary[Title] OR cardiac[Title] OR heart*[Title] OR cardiovascular[Title] OR “coronary artery disease” [Title] OR CAD[Title] OR myocarditis[Title] OR pericarditis[Title] OR valv*[Title] OR “valvular disease”[Title] OR atherosclerosis[Title] OR thrombosis[Title] OR arrhythmia[Title] OR dysrhythmia[Title] OR “Pulmonary manifestations” [Title] OR lung[Title] OR pulmon*[Title] OR pleuritis[Title] OR “interstitial lung disease” [Title] OR “pulmonary hypertension” [Title])
**Neuropsychiatric lupus**	((Systemic Lupus Erythematosus) OR SLE [Title] OR DLE[Title] OR "lupus"[Title] OR SCLE[Title] OR CLE[Title]) **AND** (“Neuropsychiatric lupus”[Title] OR psychiatric[Title] OR “CNS lupus”[Title] OR “neuropsychiatric SLE”[Title] OR “Neurological symptom*”[Title])

Supplementary Table 1. Complete Voting Results for Experts' Statements and Recommendations on Pharmacologic Treatment of SLE.

Statement/Recommendation	Poll Option	Count	Results	Percentage of Agreement
**Glucocorticoids/Steroids**				
**Whenever a high dose of steroids is needed, we recommend pulses of intravenous methylprednisolone (usually 250–1000 mg per day, for 1–3 days) to provide immediate therapeutic effect and enable the use of a lower starting dose of oral GC to minimize exposure to steroids.**	Strongly agree	3	33%	100%
	Agree	6	67%	
	Neutral	0	0%	
	Disagree	0	0%	
	Strongly disagree	0	0%	
**For chronic maintenance treatment, GC should be minimized to less than 5 mg/day (prednisone equivalent) and, when possible, withdrawn.**	Strongly agree	4	44%	100%
	Agree	5	56%	
	Neutral	0	0%	
	Disagree	0	0%	
	Strongly disagree	0	0%	
**GC withdrawal is an achievable goal in SLE and may be attempted after remission or LLDAS to protect the patient from disease flares.**	Strongly agree	3	33%	77%
	Agree	4	44%	
	Neutral	2	22%	
	Disagree	0	0%	
	Strongly disagree	0	0%	
**Despite the benefits supporting GC withdrawal, close observation after withdrawal is required and advisable due to the risk of a flare-up**	Strongly agree	5	56%	89%
	Agree	3	33%	
	Neutral	1	11%	
	Disagree	0	0%	
	Strongly disagree	0	0%	
**Prompt initiation of immunomodulatory agents can expedite the tapering/discontinuation of GC**	Strongly agree	5	56%	89%
	Agree	3	33%	
	Neutral	1	11%	
	Disagree	0	0%	
	Strongly disagree	0	0%	
**Hydroxychloroquine**				
**Previously, the maximum dose of HCQ was 5 mg/kg/real BW, while recent evidence supports**	Strongly agree	3	33%	100%
	Agree	6	67%	

**higher dosing (up to 400 mg/day, regardless of the BW).**	Neutral	0	0%	
	Disagree	0	0%	
	Strongly disagree	0	0%	
**The current evidence regarding the impact of HCQ dose tapering on the short-term and midterm outcomes in SLE is controversial. Therefore, the decision of HCQ maintenance or reduction should be personalized according to different subgroups of patients.**	Strongly agree	4	44%	88%
	Agree	4	44%	
	Neutral	1	11%	
	Disagree	0	0%	
	Strongly disagree	0	0%	
**In patients who are using HCQ dose higher than 5 mg/kg/real BW or have renal impairment, ophthalmological screening (by visual fields examination and/or spectral domain-optical coherence tomography) should be performed on annual basis.**	Strongly agree	3	33%	77%
	Agree	4	44%	
	Neutral	2	22%	
	Disagree	0	0%	
	Strongly disagree	0	0%	
**Immunosuppressive/Cytotoxic agents**				
**The choice of immunosuppressive/cytotoxic agents depends on prevailing disease manifestation(s), patient age and childbearing potential, and safety concerns.**	Strongly agree	8	89%	100%
	Agree	1	11%	
	Neutral	0	0%	
	Disagree	0	0%	
	Strongly disagree	0	0%	
**MTX and AZA should be considered in patients with poor symptom control after a trial with GC and HCQ or when HCQ alone is unlikely to be sufficient due to the experience gained with their use and their relatively safe profile**	Strongly agree	0	0%	100%
	Agree	9	100%	
	Neutral	0	0%	
	Disagree	0	0%	
	Strongly disagree	0	0%	
**CY can be considered in organ-threatening diseases (especially renal, cardiopulmonary, or neuropsychiatric) and only as rescue therapy in refractory non-major organ manifestations; due to its gonadotoxic effects, it should be used with caution in women and men of fertile age.**	Strongly agree	3	33%	100%
	Agree	6	67%	
	Neutral	0	0%	
	Disagree	0	0%	
	Strongly disagree	0	0%	
**Mycophenolate mofetil (MMF) or enteric-coated mycophenolate sodium can be used instead of cyclophosphamide (CY) and or azathioprine (AZA) in patients with active systemic lupus erythematosus.**	Strongly agree	2	22%	89%
	Agree	6	67%	
	Neutral	1	11%	
	Disagree	0	0%	

	Strongly disagree	0	0%	
**Biological agents**				
**In patients with inadequate response or intolerance to standard-of-care (combinations of HCQ, GC, and immunosuppressive agents), defined as residual disease activity not allowing tapering of glucocorticoids and/or frequent relapses, add-on treatment with belimumab and anifrolumab should be considered**	Strongly agree	0	0%	100%
	Agree	9	100%	
	Neutral	0	0%	
	Disagree	0	0%	
	Strongly disagree	0	0%	
**Belimumab or anifrolumab are considered therapeutic options for patients with SLE with mucocutaneous and/or musculoskeletal manifestations with a manageable safety profile.**	Strongly agree	6	67%	100%
	Agree	3	33%	
	Neutral	0	0%	
	Disagree	0	0%	
	Strongly disagree	0	0%	
**RTX is currently only used off-label in patients with severe renal, or extra renal (mainly hematological and neuropsychiatric) disease refractory to other IS agents and/or belimumab or in patients with contraindications to these drugs.**	Strongly agree	2	22%	100%
	Agree	7	78%	
	Neutral	0	0%	
	Disagree	0	0%	
	Strongly disagree	0	0%	
**Skin involvement**				
**For patients with cutaneous lupus erythematosus (CLE), general measures to be considered include smoking cessation and sun protection measures by applying 50 or greater sun protection factor (SPF) sunscreen in adequate amounts (2mg/cm2) at least 20 to 30 minutes before known exposure in addition to optimization of vitamin D levels.**	Strongly agree	2	22%	100%
	Agree	7	78%	
	Neutral	0	0%	
	Disagree	0	0%	
	Strongly disagree	0	0%	
**Topical agents (GC and/or CNIs) and antimalarials, with or without systemic GC, depending on the severity of skin involvement, are the recommended first-line treatment for SLE.**	Strongly agree	6	67%	100%
	Agree	3	33%	
	Neutral	0	0%	
	Disagree	0	0%	
	Strongly disagree	0	0%	
**Prolonged use of topical corticosteroids is known to cause atrophy, telangiectasia, and steroid-induced rosacea-like dermatitis. Thus, topical CNIs can exert effective steroid-sparing agents in areas at high risk of steroid complications (e.g., facial skin).**	Strongly agree	4	44%	100%
	Agree	5	56%	
	Neutral	0	0%	
	Disagree	0	0%	
	Strongly disa-	0	0%	

	gree			
**HCQ is the antimalarial of choice over chloroquine due to its multiple beneficial effects and possibly lower risk for retinal toxicity.**	Strongly agree	6	67%	100**%**
	Agree	3	33%	
	Neutral	0	0%	
	Disagree	0	0%	
	Strongly disagree	0	0%	
**MTX or other agents, such as retinoids, dapsone and MMF or EC-mycophenolic acid, can be used when first-line treatment fails to show a response in SLE.**	Strongly agree	4	44%	100**%**
	Agree	5	56%	
	Neutral	0	0%	
	Disagree	0	0%	
	Strongly disagree	0	0%	
**Belimumab and anifrolumab can be considered in resistant mucocutaneous manifestations of SLE after the failure of immunosuppressive therapy.**	Strongly agree	6	67%	100**%**
	Agree	3	33%	
	Neutral	0	0%	
	Disagree	0	0%	
	Strongly disagree	0	0%	
**Gaps and challenges regarding the pharmacologic treatment of SLE in KSA**				
**Medication adherence is not optimal in SLE patients, including Saudi populations. A routine review of treatment compliance during each visit may ensure medication adherence.**	Strongly agree	6	67%	100**%**
	Agree	3	33%	
	Neutral	0	0%	
	Disagree	0	0%	
	Strongly disagree	0	0%	
**Diagnostic delay is a major limitation in the management of SLE patients in Saudi Arabia and can negatively impact SLE outcomes.**	Strongly agree	1	11%	89**%**
	Agree	7	78%	
	Neutral	1	11%	
	Disagree	0	0%	
	Strongly disagree	0	0%	

Supplementary Table 2. Complete Voting Results for Experts' Statements and Recommendations on Treatment of specific conditions and disease monitoring.

Statement/Recommendation	Poll Option	Count	Results	Percentage of Agreement
**SLE flares**				
**Prevention of disease flares is an additional milestone of SLE treatment. Although a universally accepted definition is lacking, most experts agree that a flare is a measurable increase in disease activity, usually leading to a change of treatment**	Strongly agree	4	44%	100**%**
	Agree	5	56%	
	Neutral	0	0%	
	Disagree	0	0%	
	Strongly disagree	0	0%	
**Assessment of adherence to drug treatment, close monitoring, and optimization of disease control in these patients may reduce the risk of a flare**	Strongly agree	6	67%	100**%**
	Agree	3	33%	
	Neutral	0	0%	
	Disagree	0	0%	
	Strongly disagree	0	0%	
**GC withdrawal should be made with caution, especially in patients with serologically active yet clinically quiescent to avoid flare.**	Strongly agree	5	56%	100**%**
	Agree	4	44%	
	Neutral	0	0%	
	Disagree	0	0%	
	Strongly disagree	0	0%	
**Antiphospholipid antibodies (aPL) and antiphospholipid syndrome (APS)**				
**All patients with SLE should be screened at diagnosis for aPL due to the high risk of thrombotic events, adverse fetal outcomes, non-thrombotic events, and mortality.**	Strongly agree	4	44%	88%
	Agree	4	44%	
	Neutral	1	11%	
	Disagree	0	0%	
	Strongly disagree	0	0%	
**Patients with SLE with a high-risk aPL profile (persistently positive medium/high titers or multiple positivity) may receive primary prophylaxis with ASA, especially if other atherosclerotic/thrombophilia factors are present, after balancing the bleeding hazard.**	Strongly agree	2	22%	100%
	Agree	7	78%	
	Neutral	0	0%	
	Disagree	0	0%	
	Strongly disagree	0	0%	
**For secondary prevention (thrombosis, pregnancy complication/loss), the therapeutic approach should be the same as for primary antiphospholipid syndrome.**	Strongly agree	4	44%	77%
	Agree	3	33%	
	Neutral	1	11%	
	Disagree	1	11%	
	Strongly disagree	0	0%	
**Pregnancy**				
**Current evidence shows maternal and fetal complications were significantly higher in**	Strongly agree	5	56%	89%
	Agree	3	33%	

**SLE-associated pregnancies. Therefore, SLE should still be considered a high-risk factor for pregnancy.**	Neutral	1	11%	
	Disagree	0	0%	
	Strongly disagree	0	0%	
**Given the potential complications and morbidity, all pregnant women with SLE are considered high risk for adverse pregnancy outcomes and should be managed by a multidisciplinary team, ideally including a rheumatologist, an obstetrician with experience in lupus, an internist and, if indicated, a nephrologist.**	Strongly agree	2	22%	100%
	Agree	7	78%	
	Neutral	0	0%	
	Disagree	0	0%	
	Strongly disagree	0	0%	
**The status of SLE is intimately correlated with maternal and neonatal outcomes. Thus, accurate prediction of at-risk females before conception is crucial to avoid the negative impact of SLE on pregnancy outcomes.**	Strongly agree	0	0%	100%
	Agree	9	100%	
	Neutral	0	0%	
	Disagree	0	0%	
	Strongly disagree	0	0%	
**In patients desiring pregnancy, remission, or low lupus disease activity state (LLDAS) is the goal before pregnancy is attempted. Good pregnancy outcomes could be achieved in case of remission and adequately controlled disease activity before pregnancy.**	Strongly agree	2	22%	100%
	Agree	7	78%	
	Neutral	0	0%	
	Disagree	0	0%	
	Strongly disagree	0	0%	
**Preconception counseling is recommended for women with SLE and/or APS to implement appropriate preventive strategies and develop a personalized monitoring plan before and during pregnancy.**	Strongly agree	4	44%	100%
	Agree	5	56%	
	Neutral	0	0%	
	Disagree	0	0%	
	Strongly disagree	0	0%	
**Patients should be in LLDAS or remission for 4-6 months before trying to conceive on medications compatible with pregnancy. Those with moderate or severe disease activity should delay pregnancy until the disease is controlled on stable, pregnancy-compatible medications.**	Strongly agree	4	44%	100%
	Agree	5	56%	
	Neutral	0	0%	
	Disagree	0	0%	
	Strongly disagree	0	0%	
**In women with SLE, major risk factors for adverse maternal and fetal outcomes include active/flaring SLE, especially active nephritis, preeclampsia, eclampsia, history of lupus nephritis and presence of aPL/APS.**	Strongly agree	3	33%	100%
	Agree	6	67%	
	Neutral	0	0%	
	Disagree	0	0%	
	Strongly disagree	0	0%	
**HCQ is recommended preconceptionally and throughout pregnancy for patients with SLE.**	Strongly agree	5	56%	100%
	Agree	4	44%	
	Neutral	0	0%	
	Disagree	0	0%	
	Strongly disagree	0	0%	

**HCQ can improve pregnancy outcomes in SLE patients by reducing the risk of preeclampsia. All patients should be on HCQ unless contra-indicated.**	Strongly agree	6	67%	**78%**
	Agree	1	11%	
	Neutral	2	22%	
	Disagree	0	0%	
	Strongly disagree	0	0%	
**Blood pressure monitoring, using safe medications to control disease activity, especially HCQ, and limiting glucocorticoid exposure are essential measures.**	Strongly agree	4	44%	100**%**
	Agree	5	56%	
	Neutral	0	0%	
	Disagree	0	0%	
	Strongly disagree	0	0%	
**In pregnant women with SLE, assessment of disease activity, including renal function parameters and serological markers (serum C3/C4, anti-dsDNA titres), is recommended to monitor for obstetrical adverse outcomes and disease flares.**	Strongly agree	1	11%	100**%**
	Agree	8	89%	
	Neutral	0	0%	
	Disagree	0	0%	
	Strongly disagree	0	0%	
**Women with SLE at risk of preeclampsia (especially those with lupus nephritis or positive aPL) should receive LDA. In women with SLE-associated APS or primary APS, combination treatment with LDA and heparin is recommended to decrease the risk of adverse pregnancy outcomes.**	Strongly agree	0	0%	89**%**
	Agree	8	89%	
	Neutral	1	11%	
	Disagree	0	0%	
	Strongly disagree	0	0%	
**Current evidence indicates that non-steroidal anti-inflammatory drugs (NSAIDs) can be continued during the first and second trimesters.**	Strongly agree	1	11%	100**%**
	Agree	8	89%	
	Neutral	0	0%	
	Disagree	0	0%	
	Strongly disagree	0	0%	
**Moderate-to-severe flares can be managed with additional strategies, including glucocorticoids, intravenous pulse therapy, intravenous immunoglobulin, and plasmapheresis.**	Strongly agree	2	22%	89**%**
	Agree	6	67%	
	Neutral	0	0%	
	Disagree	1	11%	
	Strongly disagree	0	0%	
**Current evidence shows an increased risk of adverse pregnancy outcomes with glucocorticoid use (> 20 mg/day) especially preterm birth and low birth weight. Prednisolone/prednisone can be continued throughout pregnancy at the lowest effective dose.**	Strongly agree	1	11%	100%
	Agree	8	89%	
	Neutral	0	0%	
	Disagree	0	0%	
	Strongly disagree	0	0%	
**Mycophenolic acid, cyclophosphamide, leflunomide and methotrexate should be avoided during pregnancy due to known or possible teratogenicity.**	Strongly agree	8	89%	100%
	Agree	1	11%	
	Neutral	0	0%	
	Disagree	0	0%	

	Strongly disagree	0	0%	
**CYC should not be administered during the first trimester of pregnancy due to the risk of fetal loss. It should be reserved only for the management of severe, life-threatening, or refractory SLE manifestations during the second or third trimester.**	Strongly agree	5	56%	100%
	Agree	4	44%	
	Neutral	0	0%	
	Disagree	0	0%	
	Strongly disagree	0	0%	
**Current evidence indicates an increased rate of congenital malformation with methotrexate treatment. In a planned pregnancy, methotrexate should be withdrawn 1–3 months before pregnancy.**	Strongly agree	3	33%	89%
	Agree	5	56%	
	Neutral	1	11%	
	Disagree	0	0%	
	Strongly disagree	0	0%	
**Current evidence indicates an increased rate of congenital malformations. Cyclophosphamide must be withdrawn before a planned pregnancy.**	Strongly agree	6	67%	100%
	Agree	3	33%	
	Neutral	0	0%	
	Disagree	0	0%	
	Strongly disagree	0	0%	
**HCQ, oral glucocorticoids, azathioprine, ciclosporin A and tacrolimus can be used to prevent or manage SLE flares during pregnancy.**	Strongly agree	1	11%	100%
	Agree	8	89%	
	Neutral	0	0%	
	Disagree	0	0%	
	Strongly disagree	0	0%	
**Patients who responded to initial treatment with MPA should remain on MPA unless the pregnancy is contemplated, in which case they should switch to AZA at least six weeks prior to conception.**	Strongly agree	1	11%	100%
	Agree	8	89%	
	Neutral	0	0%	
	Disagree	0	0%	
	Strongly disagree	0	0%	
**Mycophenolate should be stopped six weeks respectively prior to conception. Enteric-coated mycophenolate sodium has a teratogenic potential, which poses a limitation towards universal recommendation in women of reproductive age with non-renal manifestations.**	Strongly agree	1	11%	89%
	Agree	7	78%	
	Neutral	1	11%	
	Disagree	0	0%	
	Strongly disagree	0	0%	
**Preliminary data about the safety of Belimumab during pregnancy is reassuring; nevertheless, more data are needed. Using such agents in pregnant SLE patients should be approached with caution and close monitoring.**	Strongly agree	5	56%	89%
	Agree	3	33%	
	Neutral	0	0%	
	Disagree	1	11%	
	Strongly disagree	0	0%	
**Thalidomide is effective in various subtypes of cutaneous disease. Due to its strict contraindi-**	Strongly agree	0	0%	100%
	Agree	9	100%	

**cation in pregnancy, the risk for irreversible polyneuropathy, and the frequent relapses of drug discontinuation, it should be considered only as a 'rescue' therapy in patients who have failed multiple previous agents.**	Neutral	0	0%	
	Disagree	0	0%	
	Strongly disagree	0	0%	
**Monitoring and optimal treatment targets**				
**aPL, Anti-Ro, and anti-La antibodies should be checked prior to pregnancy.**	Strongly agree	2	22%	78%
	Agree	5	56%	
	Neutral	2	22%	
	Disagree	0	0%	
	Strongly disagree	0	0%	
**Patients with lupus are at increased risk of comorbidities, such as atherosclerotic disease, osteoporosis, avascular necrosis, malignancy, and infection. Management of modifiable risk factors, including hypertension, dyslipidemia, diabetes, high BMI, and smoking, should be reviewed.**	Strongly agree	5	56%	100%
	Agree	4	44%	
	Neutral	0	0%	
	Disagree	0	0%	
	Strongly disagree	0	0%	
**Immunosuppressive therapy and hydroxychloroquine may lead to toxicities. Close monitoring of drugs by regular laboratory tests and clinical assessment should be performed following drug monitoring guidelines.**	Strongly agree	2	22%	100%
	Agree	7	78%	
	Neutral	0	0%	
	Disagree	0	0%	
	Strongly disagree	0	0%	
**Treatment in SLE should aim at remission or, if this state cannot be achieved, at low disease activity in all organ systems.**	Strongly agree	8	89%	100%
	Agree	1	11%	
	Neutral	0	0%	
	Disagree	0	0%	
	Strongly disagree	0	0%	
**Data regarding the optimal duration and timing of therapy discontinuation in renal and extrarenal disease are scarce. Gradual withdrawal of immunosuppressive drugs can be attempted after at least 3 to 5 years of therapy in complete clinical response. Hydroxychloroquine should be continued long-term.**	Strongly agree	1	11%	100%
	Agree	8	89%	
	Neutral	0	0%	
	Disagree	0	0%	
	Strongly disagree	0	0%	

Supplementary Table 3. Complete Voting Results for Experts' Statements and Recommendations on Comorbidities, adjunct therapy, and specific manifestations of SLE

Statement/Recommendation	Poll Option	Count	Results	Percentage of Agreement
**Most common comorbidities in SLE**				
**SLE patients have a higher risk of CVD compared to the general population, with the highest risk among younger**	Strongly agree	2	22%	**89%**
	Agree	6	67%	
	Neutral	1	11%	
	Disagree	0	0%	
	Strongly disagree	0	0%	
**HTN, DM, and mood/cognitive disorders, particularly depression, are the most prevalent comorbidities among Saudi SLE patients.**	Strongly agree	2	22%	**100%**
	Agree	7	78%	
	Neutral	0	0%	
	Disagree	0	0%	
	Strongly disagree	0	0%	
**Other SLE-associated comorbidities include thromboembolic events such as DVT, PE, and stroke**	Strongly agree	3	33%	**100%**
	Agree	6	67%	
	Neutral	0	0%	
	Disagree	0	0%	
	Strongly disagree	0	0%	
**SLE-associated comorbidities impact patient's treatment outcomes, quality of life, and disease progression and may become severe enough to necessitate hospitalization.**	Strongly agree	6	67%	**100%**
	Agree	3	33%	
	Neutral	0	0%	
	Disagree	0	0%	
	Strongly disagree	0	0%	
**The high prevalence of multimorbidity among patients with SLE in the community advocates for multidisciplinary care to optimize clinical outcomes**	Strongly agree	0	0%	**89%**
	Agree	8	89%	
	Neutral	1	11%	
	Disagree	0	0%	
	Strongly disagree	0	0%	
**Comorbidities screening**				
**A screening for various comorbidities at SLE diagnosis is recommended to reduce organ damage, risk of complications, and risk of mortality**	Strongly agree	0	0%	**78%**
	Agree	7	78%	
	Neutral	2	22%	
	Disagree	0	0%	
	Strongly disagree	0	0%	
**Patients with SLE should adhere to general population screening recommendations, particularly for cervical cancer and cardiovascular diseases.**	Strongly agree	1	11%	**100%**
	Agree	8	89%	
	Neutral	0	0%	
	Disagree	0	0%	
	Strongly disagree	0	0%	
**Cardiovascular diseases**				
**Clinicians should be aware of the increased**	Strongly agree	2	22%	**100%**

**cardiovascular risk among patients with SLE. Therefore, non-pharmacological interventions for CVD, such as smoking cessation, avoiding sedentary lifestyles, and maintaining an optimal BMI, should be considered for all SLE patients**	Agree	7	78%	
	Neutral	0	0%	
	Disagree	0	0%	
	Strongly disagree	0	0%	
**HCQ is to be considered in all lupus cases, unless contraindicated, due to its putative athero-protective role.**	Strongly agree	2	22%	**89%**
	Agree	6	67%	
	Neutral	1	11%	
	Disagree	0	0%	
	Strongly disagree	0	0%	
**Hormone replacement therapy (HRT) is better avoided in SLE patients due to the associated increased risk of CVD and venous thromboembolism.**	Strongly agree	0	0%	**89%**
	Agree	8	89%	
	Neutral	1	11%	
	Disagree	0	0%	
	Strongly disagree	0	0%	
**The use of validated CVD risk prediction tools tends to underestimate the actual CVR in patients with SLE.**	Strongly agree	0	0%	**100%**
	Agree	9	100%	
	Neutral	0	0%	
	Disagree	0	0%	
	Strongly disagree	0	0%	
**ACEIs or ARBs (in case of intolerance) are preferred as first-line treatment for HTN in patients with SLE due to their renoprotective effects (i.e., improve serum creatinine levels and reduce proteinuria).**	Strongly agree	2	22%	**100%**
	Agree	7	78%	
	Neutral	0	0%	
	Disagree	0	0%	
	Strongly disagree	0	0%	
**If the blood pressure is not well controlled by monotherapy or in cases with concomitant pulmonary arterial hypertension, a CCB or thiazide diuretic should be added.**	Strongly agree	0	0%	**78%**
	Agree	7	78%	
	Neutral	1	11%	
	Disagree	1	11%	
	Strongly disagree	0	0%	
**Once on antihypertensive drug therapy, all SLE patients are recommended to return for monitoring and treatment adjustment at least every second month, until they achieve their BP goal.**	Strongly agree	0	0%	**78%**
	Agree	7	78%	
	Neutral	1	11%	
	Disagree	1	11%	
	Strongly disagree	0	0%	
**Osteoporosis**				
**Factors adversely impacting BMD, particularly chronic use of glucocorticoids, should be evaluated and managed.**	Strongly agree	5	55%	**100%**
	Agree	4	45%	
	Neutral	0	0%	
	Disagree	0	0%	
	Strongly disagree	0	0%	
**Osteoporosis, often due to glucocorticoid therapy, may increase the risk of bone fractures, necessitating appropriate treatment.**	Strongly agree	5	55%	**100%**
	Agree	4	45%	
	Neutral	0	0%	

	Disagree	0	0%	
	Strongly disagree	0	0%	
**To improve bone health in SLE patients, certain lifestyle changes such as weight loss, performing weight-bearing exercises, and smoking cessation should be implemented.**	Strongly agree	6	67%	**100%**
	Agree	3	33%	
	Neutral	0	0%	
	Disagree	0	0%	
	Strongly disagree	0	0%	
**Vitamin D and calcium supplementation significantly improved the BMD in vitamin D-deficient SLE patients.**	Strongly agree	2	22%	**100%**
	Agree	7	78%	
	Neutral	0	0%	
	Disagree	0	0%	
	Strongly disagree	0	0%	
**Due to the high prevalence of osteoporosis and osteopenia among Saudi SLE patients, screening for BMD is advocated, especially in high-risk patients (such as elderly and patients on chronic GC therapy).**	Strongly agree	4	44%	**100%**
	Agree	5	56%	
	Neutral	0	0%	
	Disagree	0	0%	
	Strongly disagree	0	0%	
**Vaccination**				
**Adult patients with SLE should be encouraged to receive vaccines according to Saudi national guidelines. Influenza, pneumococcal vaccination, and SHINGRIX (Zoster Vaccine Recombinant, Adjuvanted) should be considered in all SLE patients, irrespective of their treatments.**	Strongly agree	2	22%	**100%**
	Agree	7	78%	
	Neutral	0	0%	
	Disagree	0	0%	
	Strongly disagree	0	0%	
**Hematological manifestations of SLE**				
**Anemia – including hemolytic anemia and anemia of chronic disease – and leukopenia are the most manifested hematologic abnormalities in Saudi SLE patients**	Strongly agree	1	11%	**100%**
	Agree	8	89%	
	Neutral	0	0%	
	Disagree	0	0%	
	Strongly disagree	0	0%	
	Strongly disagree	0	0%	
Abbreviations (arranged alphabetically): ACEIs: Angiotensin-Converting Enzyme Inhibitors, ARBs: Angiotensin Receptor Blockers, BMD= Bone Mineral Density, BMI: Body Mass Index, BP: Blood Pressure, CCB= Calcium Channel Blocker, CKD= chronic kidney disease. CVD= Cardiovascular Disease, DM= Diabetes Mellitus, DVT= Deep Venous Thrombosis, GC: Glucocorticoid, HCQ= Hydroxychloroquine, HRT: Hormone Replacement Therapy, HTN= Hypertension, MSK: Musculoskeletal, PE= Pulmonary Embolism, SLE= Systemic Lupus Erythematosus				

DISCLAIMER: The above article has been published, as is, ahead-of-print, to provide early visibility but is not the final version. Major publication processes like copyediting, proofing, typesetting and further review are still to be done and may lead to changes in the final published version, if it is eventually published. All legal disclaimers that apply to the final published article also apply to this ahead-of-print version.
